# RNA-binding protein KHSRP promotes tumor growth and metastasis in non-small cell lung cancer

**DOI:** 10.1186/s13046-019-1479-2

**Published:** 2019-11-27

**Authors:** Mingxia Yan, Lei Sun, Jing Li, Huajian Yu, Hechun Lin, Tao Yu, Fangyu Zhao, Miaoxin Zhu, Lei Liu, Qin Geng, Hanwei Kong, Hongyu Pan, Ming Yao

**Affiliations:** 1 State Key Laboratory of Oncogenes and Related Genes, Shanghai Cancer Institute, Renji Hospital, Shanghai Jiao Tong University School of Medicine, No. 25/2200, Xietu Road, Shanghai, 200032 China; 2 Fudan University Shanghai Cancer Center, Shanghai Medical College, Fudan University, Shanghai, 200032 China

**Keywords:** KHSRP, Growth, Metastasis, IFN-α-JAK-STAT1 signaling pathway, Non-small cell lung cancer

## Abstract

**Background:**

KH-type splicing regulatory protein (KHSRP) plays an important role in cancer invasion, but the relevant mechanism is not well known. In the present study, we investigated the function and potential molecular mechanism of KHSRP in non-small cell lung cancer (NSCLC) metastasis and elucidated its clinical significance.

**Methods:**

Isobaric tags for relative and absolute quantitation and the SWATH™ approach were combined with nanoliquid chromatography-tandem mass spectrometry analysis to identify metastasis-associated nucleoproteins in NSCLC. Real-time PCR and Western blot were used to screen for metastasis-associated candidate molecules. Gene knockdown and overexpression were used to investigate their functions and molecular mechanisms in lung cancer cells. Coimmunoprecipitation (Co-IP) experiments were performed to identify the interactions between candidate molecules and their interacting proteins. Gene expression and its association with multiple clinicopathologic characteristics were analyzed by immunohistochemistry (IHC) and Western blot in human lung cancer specimens.

**Results:**

KHSRP was identified as a metastasis-associated candidate molecule. In NSCLC cell lines, knockdown of KHSRP significantly reduced lung cancer cell proliferation, migration, and invasion in vitro and in vivo, whereas overexpression of KHSRP did the opposite. Mechanistically, the protein heterogeneous nuclear ribonucleoprotein C (C1/C2) (HNRNPC) was identified to interact with KHSRP using Co-IP experiments. In NSCLC cell lines, overexpression of HNRNPC significantly promoted lung cancer cell proliferation, migration, and invasion in vitro and in vivo. KHSRP and HNRNPC may induce human lung cancer cell invasion and metastasis by activating the IFN-α-JAK-STAT1 signaling pathway. Drastically higher expression levels of KHSRP and HNRNPC were observed in lung cancer tissues compared to those in adjacent noncancerous tissues. Increased KHSRP and HNRNPC expression was significantly associated with advanced tumor stages and metastasis (both lymph node and distant). Kaplan-Meier survival analysis showed that patients with high KHSRP and HNRNPC expression levels were predicted to have the shortest survival times and to have a poor prognosis.

**Conclusions:**

KHSRP plays an important role in NSCLC metastasis and may serve as a potential prognostic marker and novel therapeutic target for lung cancer metastasis treatment.

## Background

According to statistical analysis, cancer is expected to rank as the leading cause of death and as the single most important barrier to increasing life expectancy in every country of the world in the twenty-first century. According to estimates from the World Health Organization (WHO) in 2015, cancer is the first or second leading cause of death before age 70 in 91 of 172 countries, and it ranks third or fourth in an additional 22 countries. There will be an estimated 18.1 million new cancer cases and 9.6 million cancer deaths in 2018. In both sexes combined, lung cancer is the most commonly diagnosed cancer and the leading cause of cancer death [1, 2]. Although the early diagnosis and clinical treatment of lung cancer have been greatly advanced, it cannot be ignored that most lung cancer patients are diagnosed in only the middle and late stages, the 5-year survival rate is less than 15%, and more than 90% of lung cancer patients die of metastasis [3]. Although scientists have conducted many studies on lung cancer metastasis, our understanding of lung cancer metastasis is still very limited, and the molecular mechanism underlying lung cancer metastasis is still unclear. Therefore, elucidating the molecular mechanism of NSCLC invasion and metastasis and identifying new drug targets that interfere with lung cancer metastasis are key scientific problems that need to be solved in the field of lung cancer research.

Proteomics research is a high-throughput, large-scale protein research model that uses liquid and mass spectrometry as the core technology. Proteomics is mainly the study of protein-protein interactions to clarify protein functions [4]. Currently, the most effective strategies in proteomics research are comparing the similarities and differences in all proteins or regional organelle subunits expressed in cells or tissues under different physiological and pathological conditions [5]. By using this strategy, many key protein molecules of clinical significance can be screened, and target protein molecules that are the source of drug action can be found. This strategy provides a new idea for the study of lung cancer metastasis [6].

In the present study, we analyzed differential metastatic potential lung cancer cells using isobaric tags for relative and absolute quantitation (iTRAQ) and the SWATH™ approach combined with nano liquid chromatography-tandem mass spectrometry (NanoLC-MS/MS) analysis. Small-sample clinical verification and preliminary functional experiments demonstrated that the differential expression of KHSRP (also known as far upstream element-binding protein 2, KSRP/FBP2/FUBP2) was the most significant protein in high and low metastatic cells. We focused on the roles of KHSRP in lung cancer metastasis in vitro and in vivo, identified the protein molecules that interacted with KHSRP by using Coimmunoprecipitation (Co-IP) and proteomic analyses, verified the functions and effects of the interacting proteins in promoting metastasis, elucidated the molecular mechanism of metastasis, demonstrated the potential tumor metastasis-related signaling pathway, and validated its clinical significance in a mass of lung cancer specimens.

## Methods

### Cell lines and cell culture

The human NSCLC cell lines A549, NCI-H1299, NCI-H838, NCI-H358, and NCI-H292 and human embryonic kidney HEK-293 T cells were obtained from American Type Culture Collection (ATCC, Manassas, VA). All cell lines were cultured according to ATCC protocols in medium supplemented with 10% fetal bovine serum (Biowest, South America origin), 100 u/ml penicillin (Sigma-Aldrich, UA), and 100 μg/ml streptomycin (Sigma-Aldrich, UA) at 37 °C in a humidified atmosphere containing 5% CO2.

### Nuclear protein extraction, quantification and iTRAQ labeling

The nuclear proteins of NCI-H1299 and NCI-H358 cells were extracted and quantified by BCA protein quantitative method. iTRAQ labeling was conducted using an iTRAQ Reagent 4-Plex kit (Applied Biosystems, Foster City, CA) according to the manufacturer’s protocol. Briefly, 100 μg lysate of each sample was reduced with tris- (2-carboxyethyl) phosphine and alkylated with methyl methanethiosulfonate and then digested overnight at 37 °C with trypsin (mass spectrometry grade, Promega, Madison, WI). The ratio of trypsin to protein was 1:20. The iTRAQ labeled samples were then combined according to the specified set and transferred into a new EP tube, desalted with Oasis HLB cartridges (Waters, Milford, MA) and dried in a vacuum centrifuge (Concentrator Plus, Eppendorf, Germany) [7].

### NanoLC−MS/MS analysis

A NanoLC system (NanoLC-2D Ultra, Eksigent, Dublin, CA) equipped with a Triple TOF 5600 mass spectrometer (AB SCIEX, USA) was used for analysis. Peptides were trapped on a NanoLC pre-column (Chromxp C18-LC-3 μm, size 0.35 × 0.5 mm, Eksigent, Dublin, CA) and then eluted onto an analytical column (C18-CL-120, size 0.075 × 150 mm, Eksigent, Dublin, CA). The NanoLC gradient was 5–35% Buffer B (98% ACN, 2% H2O, 0.1% FA) over 120 min at a flow rate of 300 nL/min. Full-scan MS was performed in positive ion mode with a nano-ion spray voltage of 2.2 kV. Survey scans were acquired from 350 to 1500 (m/z) with up to 40 precursors selected for MS/MS (m/z 100–1500). The collision energy (CE) for collision-induced dissociation was automatically controlled using an Information-Dependent Acquisition CE parameter script to achieve optimum fragmentation efficiency. The mass spectrometer was calibrated using beta galactosidase tryptic peptides.

### Protein identification and quantitation

Protein identification and iTRAQ quantitation were performed with ProteinPilot 4.5 software (AB SCIEX, USA). A strict unused confidence cutoff > 1.3 and more than two peptides were used as the qualification criteria. The peptide confidence level was 95%. Proteins with a fold change larger than 1.5 or less than 0.67 with a Student’s t-test *p*-value <0.05 were selected as differentially expressed proteins. The up-regulated and down-regulated proteins were analyzed by DAVID Bioinformatics Resources 6.7 (http://david.abcc.ncifcrf.gov). The up-regulated and down-regulated proteins were analyzed from the three GO terms of biological processes (BP), cellular component (CC) and molecular functions (MF), respectively.

### SWATH™ analysis and library generation

Samples were analyzed on the mass spectrometer in two phases: data-dependent acquisition (DDA) was followed by SWATH acquisition on the same sample with the same gradient conditions used. The detailed parameter setting and library generation methods could be referred to the reference [8]. We selected proteins with at least two peptides identified for relative quantitation analysis. With three technical replicates for each sample, analysis of relative quantitation was performed by t-test analysis. The protein levels with a *p*-value less than 0.05 were considered significantly different in our experiment.

### RNA extraction and real-time polymerase chain reaction assay

Total RNA was extracted from the lung cancer cells and tissues using TRIzol Reagent (Invitrogen, Carlsbad, CA) according to the manufacturer’s protocol. cDNA was synthesized by random primers and the PrimeScript RT Reagent Kit (Takara, Dalian, China). The primer sequences for real-time PCR are shown in Additional file 1: Table S1. Real-time polymerase chain reaction (qPCR) was performed using SYBR Premix Ex Tag (Takara, Dalian, China). The PCR conditions were as follows: 95 °C for 15 s followed by 40 cycles of 95 °C for 5 s and 60 °C for 30 s. The PCR primers used are listed in Table S1. β-actin was used as the internal control.

### Protein extraction and Western blotting

Cell and tissue proteins were extracted using T-PER® Protein Extraction Reagent (Thermo Scientific, USA) with a phosphatase inhibitor cocktail (Roche Applied Science, Switzerland) and a proteinase inhibitor cocktail (Roche Applied Science, Switzerland). The protein concentrations were determined using the Pierce™ BCA Protein Assay Kit (Thermo Scientific, USA). Proteins were separated by SDS-PAGE gels and transferred to nitrocellulose (NC) filter membranes (Millipore, Massachusetts, USA) or polyvinylidene fluoride (PVDF) membranes (Millipore, Massachusetts, USA). The membranes were incubated with primary antibodies overnight at 4 °C and probed with secondary antibodies at room temperature for 1~2 h. The following antibodies were used: anti-KHSRP (Abgent, 1:500), anti-HNRNPC (Sigma-Aldrich, 1:1000), anti-IFN-α (Santa Cruz, 1:500), anti-JAK1 (Santa Cruz, 1:500), anti-JAK2 (Santa Cruz, 1:500), anti-p-Stat1 (Sigma-Aldrich, 1:1000), anti-Stat1 (Sigma-Aldrich, 1:1000), anti-p-Stat3 (Sigma-Aldrich, 1:1000), anti-p-Akt (CST, 1:1000), anti-p-Erk (CST, 1:1000) and anti-α-Tubulin (CST, 1:1000).

### Cell transient transfections

Small-interfering RNA (siRNA) oligonucleotides for KHSRP and HNRNPC were designed and synthesized by RiboBio (Guangzhou, China). The primer sequences for the siRNAs are shown in Additional file 1: Table S2. Transient transfection was performed using Lipofectamine 2000 Reagent (Invitrogen, Carlsbad, USA) according to the manufacturer’s instructions. After transfection for 48 h, the cells were used for functional assays, including migration, invasion, RNA extraction, and Western blotting.

### Cell viability assay

Cells were seeded in 96-well plates at 1 × 10^3^ cells per well and cultured in a final volume of 100 μl of culture medium supplemented with 10% FBS. According to the manufacturer’s instructions, 10 μl of Cell Counting Kit-8 (CCK-8, Dojindo, Kumamoto, Japan) was added to each well, and the mixture was incubated at 37 °C for 2 h. The absorbance was measured at 450 nm.

### Cell migration and invasion assays

Cell migration and invasion assays were performed in 24-well plates with 8-μm-pore size chamber inserts (BD Biosciences, New Jersey, USA). For the migration assays, 5 × 10^4^ cells in 200 μl of serum-free culture medium were seeded into each well of the upper chamber with the noncoated membrane, and 800 μl of medium supplemented with 10% FBS was added to the lower chamber. For invasion assays, 1 × 10^5^ cells in 200 μl of serum-free culture medium were seeded into each well of the upper chamber with the Matrigel-coated membrane, and 800 μl of medium supplemented with 10% fetal bovine serum (FBS) was added to the lower chamber. The cells that migrated through the membrane were fixed with 100% methanol, stained with 0.1% crystal violet for 30 min, imaged and counted under a light microscope (Olympus, Japan).

### Wound-healing assays

For the wound-healing assay, cells were seeded into 24-well plates and grown to approximately 90% confluence. The monolayers were scratched with a 200 μl sterile pipette tip. The cells were rinsed with phosphate-buffered saline and cultured in Dulbecco’s Modified Eagle’s Medium (DMEM) (or Roswell Park Memorial Institute 1640 medium, RPMI 1640) supplemented with 1% fetal bovine serum. The wounded monolayers were photographed at 0 h and 24 h after the wounds were made.

### In vivo tumor growth and metastasis

All animal experiments were approved by the Animal Ethics Committee of the Shanghai Cancer Institute. Six- to eight-week-old female BALB/c-nu/nu mice were bred by Shanghai Cancer Institute (Shanghai, China) and housed in specific pathogen-free (SPF) conditions in a laboratory animal facility.

For the in vivo xenograft assays, 3 × 10^6^ A549 cells stably expressing shKHSRP or the negative control and 2 × 10^6^ NCI-H292 cells stably expressing KHSRP or the lentiviral vector were separately subcutaneously inoculated into the dorsal right flanks of the nude mice (6 per group). The tumor size was measured two times every week. The tumor volume (V) was measured by calipers and calculated according to the following formula: (length × width × width)/2. After eight or 10 weeks, the mice were sacrificed, and the tumors were harvested at necropsy and fixed in 10% neutral PB-buffered formalin. The fixed tumors were stained with hematoxylin and eosin (H & E).

For the in vivo metastasis assays, 3 × 10^6^ A549 cells stably expressing shKHSRP or the negative control (8 per group) and 2 × 10^6^ NCI-H292 cells stably expressing KHSRP or the lentiviral vector (10 per group) were separately injected into the lateral tail veins of nude mice. After eight or 10 weeks, the mice were sacrificed, and their lungs were harvested at necropsy and fixed in 10% neutral PB-buffered formalin. The fixed lung tissues were stained with H & E and analyzed for the presence of metastasis.

### Luciferase imaging and GFP imaging

3 × 10^6^ A549 cells stably expressing shKHSRP or the negative control (8 per group) were separately injected into the lateral tail veins of nude mice. After eight or 10 weeks, the mice were sacrificed. We used a Berthold LB983 NightOwl System (Berthold, Bad Wildbad, Germany) to monitor the tumour lung metastasis. For ex vivo biofluorescence imaging (ex vivo BFI), mice lungs were excised and placed in the chamber of the NightOwl LB 983 Molecular Light Imager and imaged.

### Coimmunoprecipitation and mass spectrometry

HEK-293 T cells with Flag- and HA-tagged-KHSRP or control were routine cultured. When the cells fullness reached more than 90%, the cells were scraped off directly with a cell scraper using immunoprecipitation lysis buffer (Beyotime Institute of Biotechnology, Shanghai, China) with protease and protein phosphatase inhibitors (Roche Applied Science, Switzerland). Three milligrams of protein were incubated with 30 μl of protein A/G magnetic beads (Millipore, Massachusetts, USA) for 2 h. The beads were removed, and 12 μl of the primary antibody (Flag, HA, KHSRP or HNRNPC) or isotype IgG was added to the supernatant at 4 °C overnight with gentle mixing on a rocking platform to capture the fusion proteins. Then, 40 μl of protein A/G beads was added to each immunoprecipitation mixture for 4 h. The magnetic beads were collected by placing the tube in the appropriate magnetic separator. The beads were washed three times with cooled IP lysis buffer to remove the nonspecifically bound proteins. The bound fusion proteins were eluted from the beads and denatured by boiling for Western blot analysis. The bound fusion proteins were separated by SDS-PAGE and stained with a Silver Staining Kit (Beyotime Biotechnology, Shanghai, China). The specific bands were identified, and the peptides were digested with trypsin. The peptides were then analyzed on an Orbitrap Fusion mass spectrometer (Thermo Fisher, Waltham, USA). Protein identification was performed using Protein Pilot 4.5 (AB Sciex, Texas, USA) [9].

### Human clinical specimens and tissue microarray

Fresh human non-small cell lung cancer tissues and matched adjacent noncancerous tissues for real-time PCR and Western blot analyses were collected from the Department of Lung Cancer at Shanghai Chest Hospital affiliated with Shanghai Jiao-tong University and the Department of Cancer at Huashan Hospital affiliated with Fudan University, Shanghai, China between 2011 and 2018. During the operation, human surgical specimens were immediately frozen in liquid nitrogen and stored at −80 °C for further investigation [10]. All of the tissue specimens for this study were obtained with patient informed consent. The study was approved by the Ethics Committee of Shanghai Jiao-tong University and the Ethics Committee of Fudan University.

A tissue microarray containing 75 paired NSCLC tissues and matched adjacent noncancerous tissues was purchased from Shanghai Biochip Co., Ltd. (Shanghai, China). Immunohistochemical staining was performed to detect the expression of KHSRP and HNRNPC in NSCLC tissues and matched noncancerous tissues. The average gray value of the image was used as a quantitative evaluation of the expression level using Image-Pro Plus 6.0 software.

### Statistical analysis

Differences among variables were assessed by χ2 analysis or two-tailed Student’s t-tests. Kaplan-Meier analysis was used to assess survival. Difference in survival were analyzed using the log-rank test. The correlation of KHSRP and HNRNPC expression was examined by Spearman’s correlation test. Data are presented as the mean ± standard deviation (SD) or the mean ± standard error of the mean (SEM). Differences were considered statistically significant at *P* < 0.05.

## Results

### Identification of KHSRP as a candidate tumor metastatic-related nuclear protein in NSCLC

**Fig. 1:**
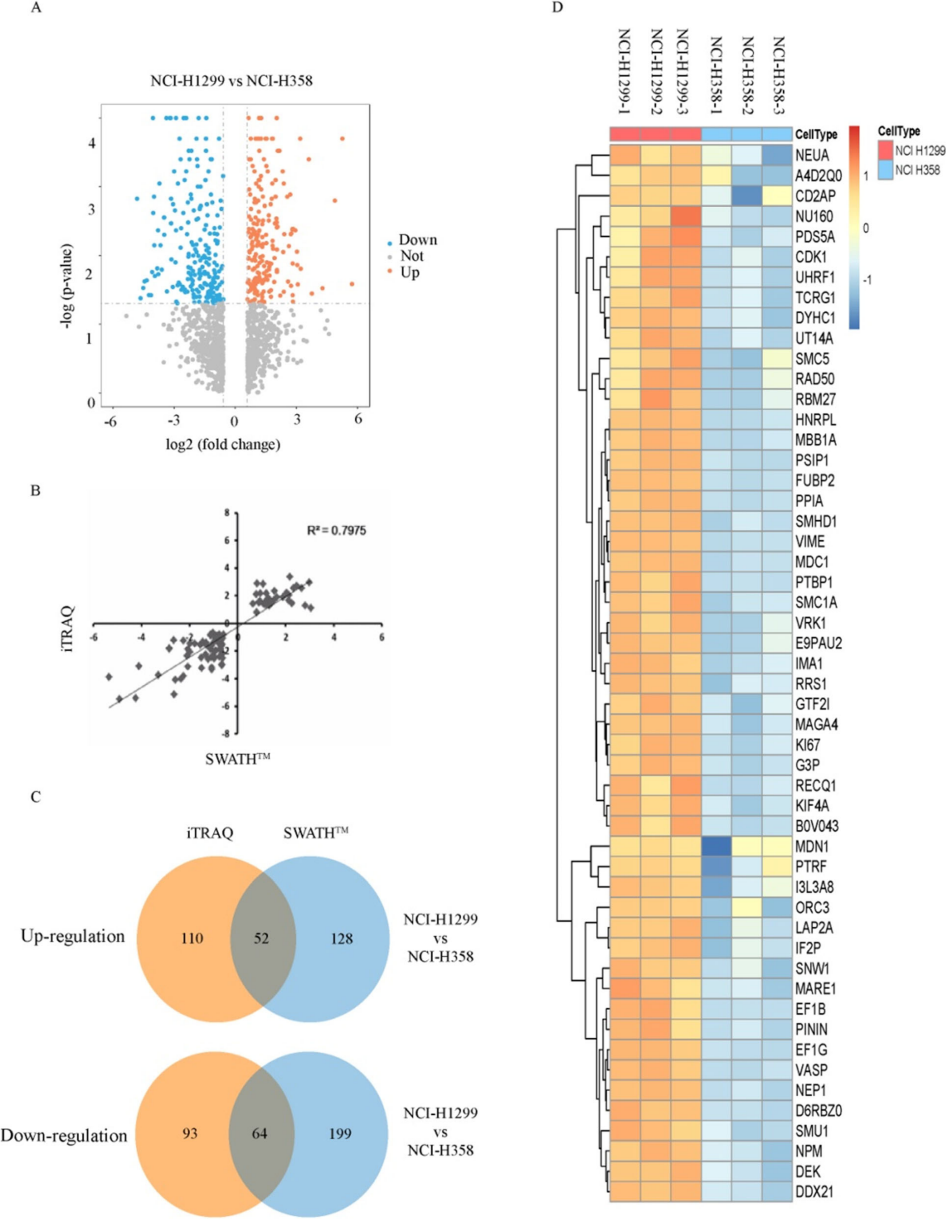
Identification of tumor metastatic-related nuclear proteins by using the iTRAQ-labeling approach and the SWATH™ approach in NSCLC. **a** The volcano map shows the nuclear protein identified by using the iTRAQ-labeling approach. **b** Scatter plot analysis of the quantified proteins was performed using the iTRAQ-labeling and SWATH™ approaches. **c** The Venn diagram shows the number of proteins intersected by two quantitative proteomics approaches. **d** The heatmap shows the upregulated differential expression of nuclear proteins determined using the SWATH™ approach

To discover nuclear proteins potentially associated with cancer metastasis, we used human lung cancer high metastatic NCI-H1299 cells and low metastatic NCI-H358 cells as experimental materials in combination with iTRAQ and SWATH™, two proteomics methods, to analyze and identify differentially expressed nuclear proteins. In the iTRAQ experiments, a total of 4324/4353 proteins (global FDR < 1%) were identified using Protein Pilot 4.5 in two technical replicates. An unused protein score > 1.3 and peptides ≥2 were used as criteria, and 3987/3896 proteins were identified; 3859/3796 proteins were quantified. We identified 162 upregulated proteins and 157 downregulated differentially expressed nucleoproteins (Fig. 1a). In the SWATH™ experiments, a total of 1129 proteins (global FDR < 1%) were identified, and 867 proteins were quantified with peptides ≥2 and *P* < 0.05 in three technical replicates. We identified 180 upregulated and 263 downregulated differentially expressed nucleoproteins. We observed a similar distribution pattern at the protein level in the two approaches, and most quantified proteins were distributed close to 0 and within a ratio range of ±2 Log_2_ (Additional file 1: Figure S1A). The consistency and correlation between the quantitative iTRAQ and SWATH™ methods were good (R^2^ = 0.7975) (Fig. 1b). Through bioinformatics analyses, the experimental data for the differentially expressed nuclear proteins obtained by a pair of high and low metastatic cells and two protein quantitative methods were integrated. After the intersection, 116 lung cancer metastasis-related differentially expressed nucleoproteins were identified, including 52 upregulated proteins and 64 downregulated proteins (Fig. 1c, d; Additional file 1: Table S3, Additional file 1: Table S4). Gene Ontology (GO) analysis revealed that these proteins are mainly involved in DNA metabolism, mRNA metabolism, RNA cleavage, DNA processes, RNA processes and other functions. The molecular functions mainly included nucleotide binding, ribonucleotide binding, ATP binding, etc. (Additional file 1: Figure S1B, C, D). The mass spectrometry proteomics data have been deposited to the ProteomeXchange Consortium (http://proteomecentral.proteomexchange.org) via the iProX partner repository with the dataset identifier PXD015861.

According to the literature reports, we selected the upregulated proteins which had been reported in metastasis but not in NSCLC metastasis or reported in cancer but not in metastasis or not reported at all in cancer. Finally, 9 potential NSCLC metastasis-associated proteins, including SMC5, PSIP1, KHSRP, VRK1, GTF21, PTRF, SNW1, VASP, DDX21, were selected to further validate the mass spectrometry results (Additional file 1: Figure S2A). In order to observe the expression of these proteins, the expression levels of candidate proteins were determined by qRT-PCR and Western blot in NCI-H1299, A549, NCI-H358 and NCI-H292 cell lines. The results showed that KHSRP, PSIP1 and VASP were higher expressed in the highly metastatic potential NSCLC cells (NCI-H1299 and A549) than in the low metastatic potential cells (NCI-H358 and NCI-H292) (Additional file 1: Figure S2B, C). To investigate whether KHSRP, PSIP1 and VASP were involved in human NSCLC metastasis progress, siRNA was used to knock down the expression of these genes in A549 cell line (Additional file 1: Figure S3A), and then the in vitro invasion and migration abilities of cells transfected with siRNAs were evaluated using transwell assays (Additional file 1: Figure S3B). We observed that knockdown of KHSRP genes could significantly inhibit the migratory and invasive abilities of NSCLC cells. These results suggested that the KHSRP might be crucial in the metastasis of NSCLC cells.

### KHSRP promoted NSCLC cell proliferation, migration and invasion

**Fig. 2:**
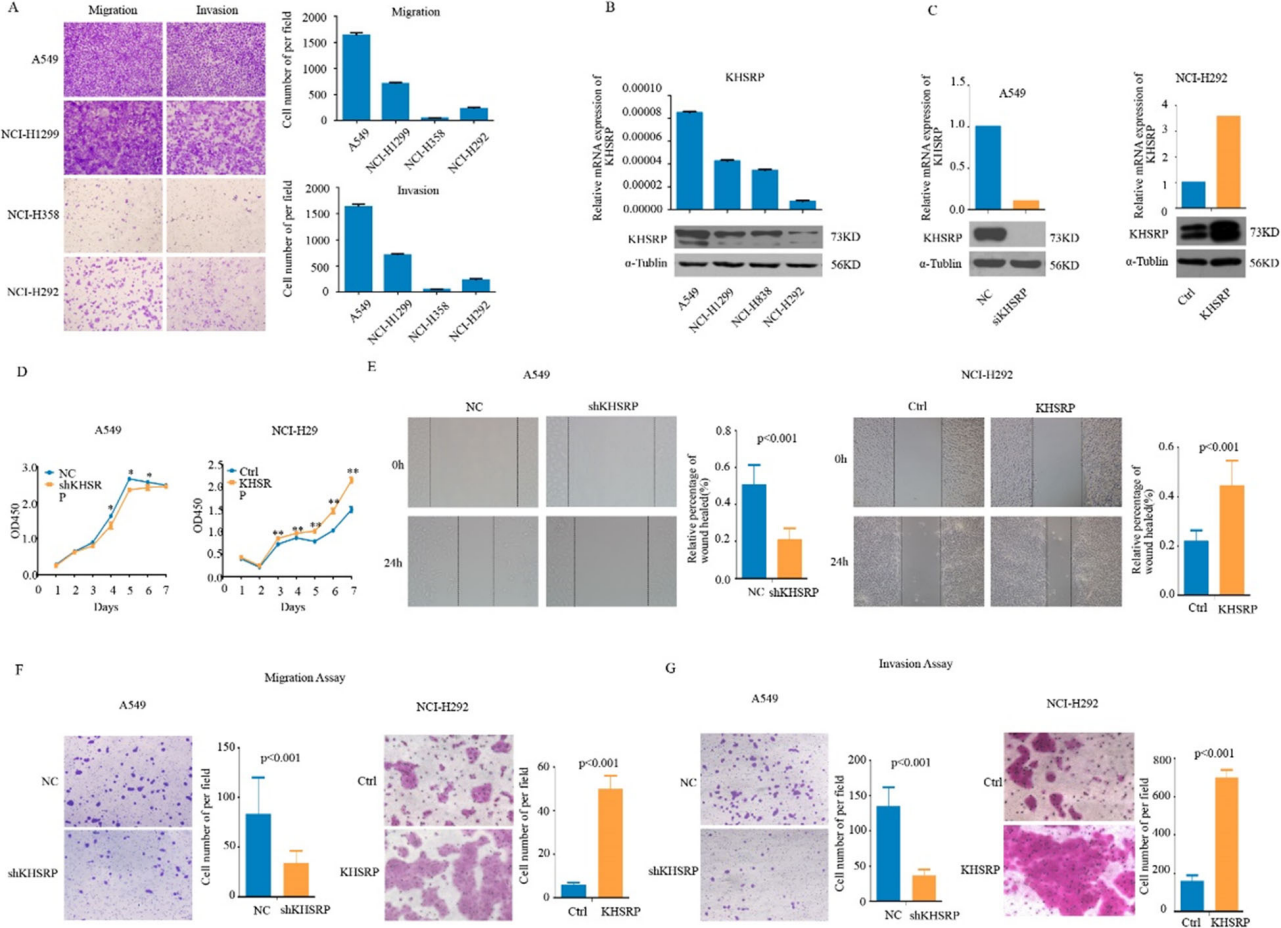
KHSRP promoted NSCLC cell proliferation, migration and invasion. **a** The migration and invasion abilities of the four human lung cancer cell lines were assessed by Transwell assays. **b** The levels of KHSRP expression in 4 human NSCLC cell lines were measured by real-time PCR and Western blotting. **c** The mRNA and protein levels of KHSRP in KHSRP knockdown and negative control A549 cells as well as in KHSRP overexpression and control NCI-H292 cells were determined by real-time PCR and Western blotting analyses, respectively. **d** The effect of KHSRP on cell proliferation was evaluated by a CCK-8 assay in A549 and NCI-H292 cells. **e** Wound-healing assays were performed in A549 and NCI-H292 cells. **f** The effect of KHSRP on cell migration was assessed by Transwell migration assays in A549 and NCI-H292 cells. **g** The effect of KHSRP on cell invasion was assessed by Matrigel invasion assays in A549 and NCI-H292 cells

To investigate the functional roles of KHSRP in NSCLC cells, we first assessed the migration and invasion abilities of the four human lung cancer cell lines. The result showed that A549 and NCI-H1299 cell lines had higher potential to metastasis than NCI-H358 and NCI-H292 cell lines (Fig. 2a) [11]. We detected the expression levels of KHSRP in a panel of 4 NSCLC cell lines (A549, NCI-H1299, NCI-H838 and NCI-H292) by real-time PCR and Western blotting (Fig. 2b). The A549 cell line showed high endogenous KHSRP expression, while the NCI-H292 cell line showed low endogenous KHSRP expression, indicating that KHSRP expression was much higher in the highly metastatic NSCLC cell lines than in the NSCLC cell lines with low metastatic potential. Therefore, we selected A549 cells for KHSRP knockdown and NCI-H292 cells for KHSRP overexpression.

We established stable models of KHSRP knockdown in A549 cell lines and stable models of KHSRP overexpression in the NCI-H292 cell line (Fig. 2c). The CCK-8 assay showed that KHSRP knockdown substantially reduced the proliferation ability of A549 cells, whereas the overexpression of KHSRP significantly enhanced the proliferation ability of NCI-H292 cells (Fig. 2d). A wound-healing assay showed that the knockdown of KHSRP decreased A549 cell migration at the edge of exposed regions, whereas the overexpression of KHSRP enhanced NCI-H292 cell migration at the edge of exposed regions (Fig. 2e). Furthermore, the migration and Matrigel invasion assays in vitro were performed with A549 and NCI-H292 cells. The results showed that the knockdown of KHSRP in A549 cells significantly inhibited the cell migration and invasion potential, whereas the overexpression of KHSRP in NCI-H292 cells markedly promoted the cell migration (*P* < 0.001, Fig. 2f) and invasion potential (*P* < 0.001, Fig. 2g). These results indicated that the knockdown of KHSRP significantly inhibited the proliferation, migration, and invasion, whereas the overexpression of KHSRP significantly promoted the proliferation, migration, and invasion of NSCLC cells in vitro.

### KHSRP promoted the xenograft tumor growth and metastatic potential of NSCLC cells in vivo

**Fig. 3:**
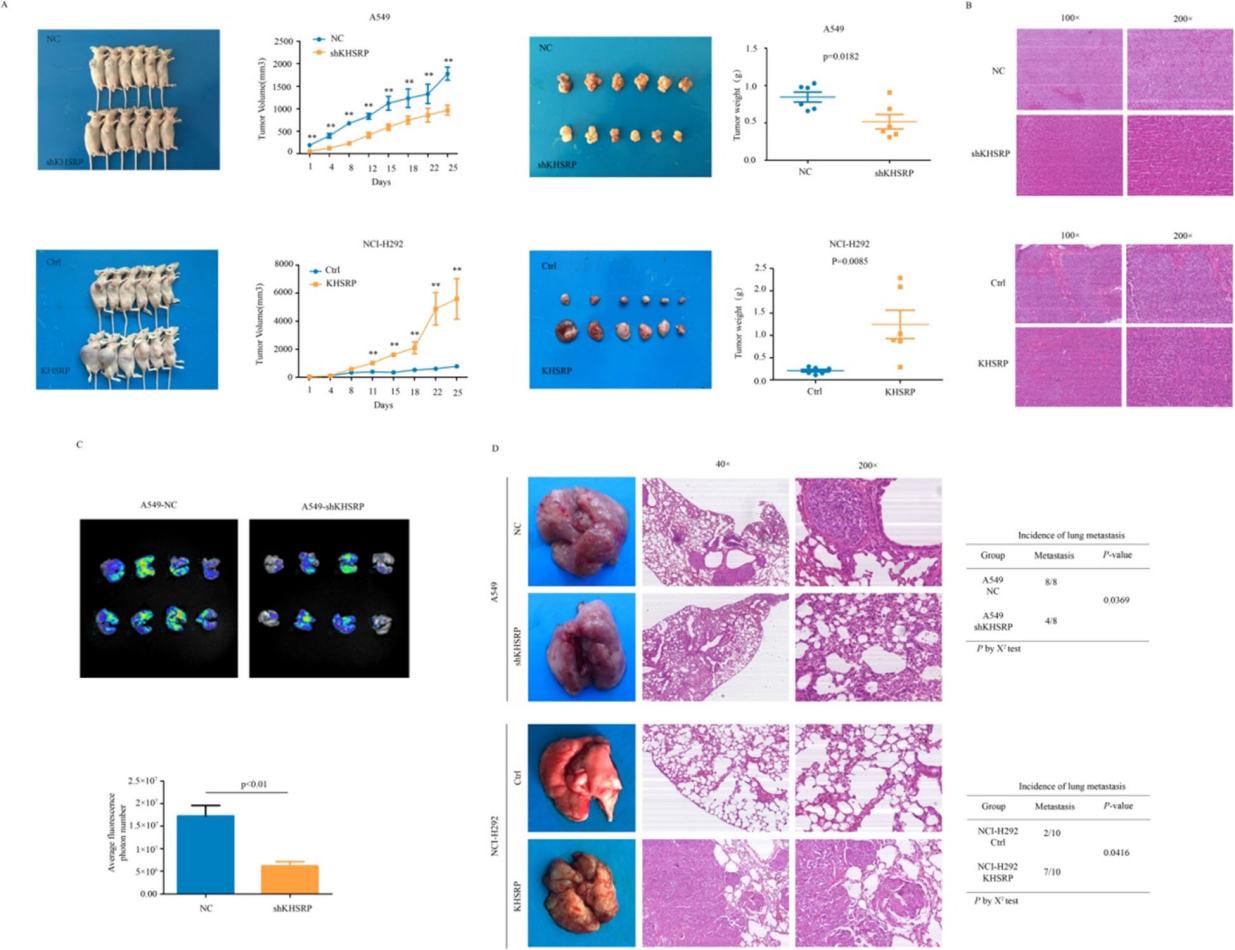
KHSRP promoted the xenograft tumor growth and metastatic potential of NSCLC cells in vivo. **a** Representative gross photos of mice and the subcutaneous tumor growth curves of KHSRP knockdown A549 cells and KHSRP overexpression NCI-H292 cells in nude mice are shown. **b** The xenograft tumors of mice were confirmed by H&E staining. **c** The in vivo imaging of animals showed the intensity and area of the luciferase expression of KHSRP in A549 cells. **d** Representative gross photos of mouse lungs and images of the histological inspection of mouse lungs for the presence of microscopic lesions 8 weeks after tail vein injection are shown. Statistical analysis was performed using Student’s t-test or the χ2 test. The error bars represent S.E.M. *P < 0.05; **P < 0.01; ***P < 0.001

To assess whether KHSRP could impact tumorigenic capacity in vivo, a xenograft tumor mouse model was established by subcutaneously injecting A549-NC, A549-shKHSRP, NCI-H292-Vector and NCI-H292-KHSRP cells into the right dorsal flanks of nude mice. After 25 days, the tumor volume of the KHSRP interference group was smaller than that of the negative control group (*P* < 0.01), and the tumor volume of the KHSRP overexpression group was significantly larger than that of the control group (*P* < 0.01). At the end of the experiments, the xenograft tumors were isolated, and their weights were measured. The tumor weights of the KHSRP stable interference group were smaller than those of the negative control group (*P* < 0.05); in addition, the tumor weights of the overexpression group were higher than those of the control group (*P* < 0.01), and this was consistent with the results of the tumor volume change (Fig. 3a, b).

To evaluate whether KHSRP could promote tumor metastatic capacity in vivo, we injected A549-NC and A549-shKHSRP cells into the lateral tail veins of nude mice (eight mice per group). After 8 weeks, the in vivo imaging of animals showed that the intensity and area of luciferase expression in the interference group were significantly lower than those in the control group (Fig. 3c); at the same time, the knockdown of KHSRP significantly decreased the number of in vivo metastatic lung nodules compared to that in the negative control group (*n* = 8, *P* < 0.05). In contrast, the stable overexpression of KHSRP significantly increased the number of metastatic lung nodules (*n* = 10, *P* < 0.05). Metastatic nodules on the surfaces of mouse lungs were confirmed by H & E staining (Fig. 3d). Therefore, our data demonstrate that high KHSRP expression enhanced tumor growth and metastasis in vivo, which was consistent with our in vitro findings.

### KHSRP physically interacted with HNRNPC in NSCLC cells

**Fig. 4:**
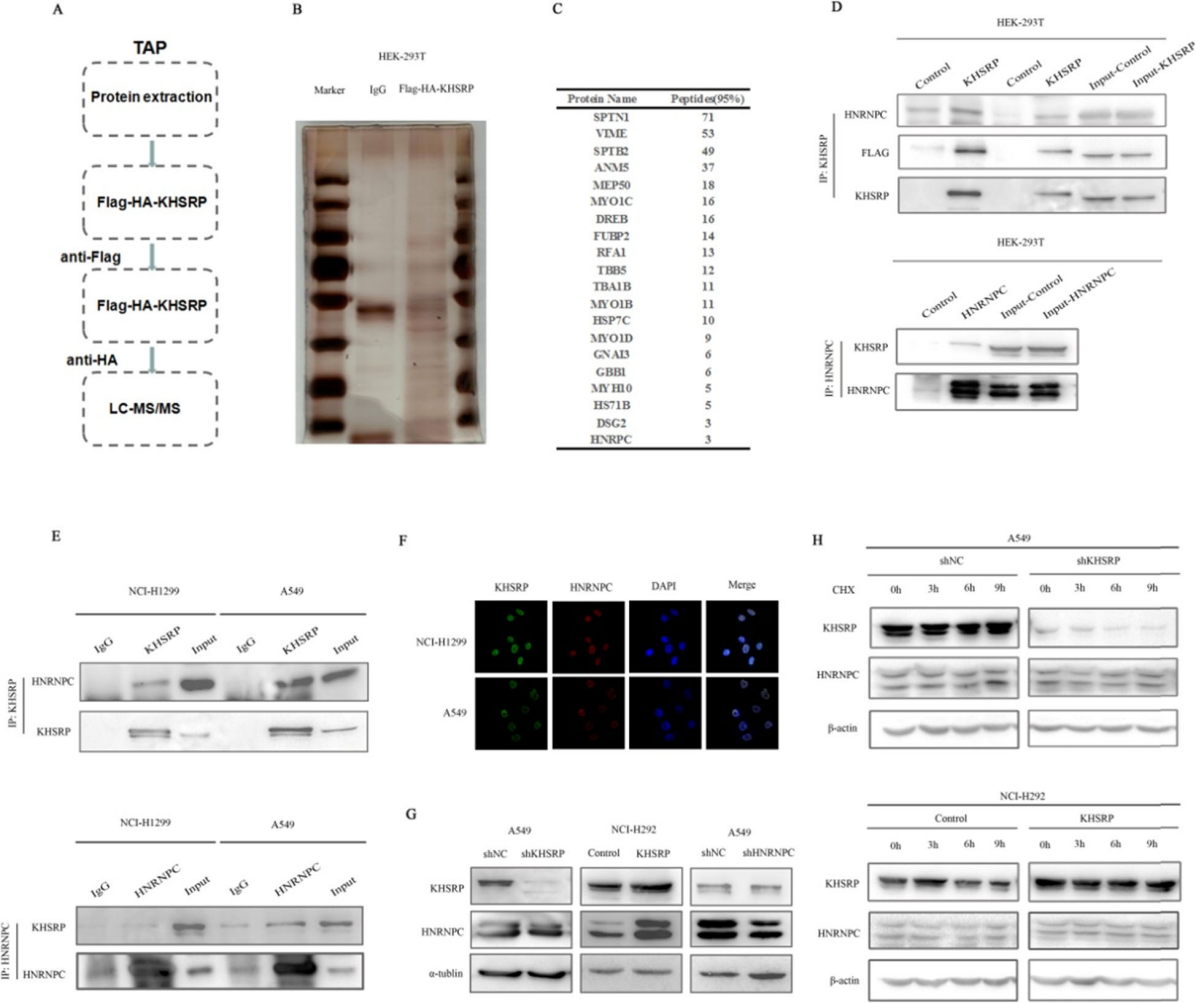
KHSRP physically interacted with HNRNPC in NSCLC cells. **a** A diagram showing tandem affinity purification (TAP) and mass spectrometry analyses of KHSRP-associated proteins. **b** Silver staining was used to detect immunoprecipitated Flag-HA-KHSRP-binding proteins. **c** A list of representative proteins that interact with the KHSRP protein is shown. **d** The interaction between KHSRP and HNRNPC detected by mass spectrometry analysis was verified by immunoprecipitation in HEK293T cells transfected with plasmids encoding KHSRP or HNRNPC. **e** The interaction between endogenous KHSRP and HNRNPC was detected by immunoprecipitation in NCI-H1299 and A549 cells. **f** Immunofluorescence staining was used to detect the colocalization and coexpression of the KHSRP and HNRNPC proteins in the nucleus. **g** The regulatory relationship between KHSRP and HNRNPC was verified by Western blotting. **h** A549 cells with KHSRP knockdown and NCI-H292 cells overexpressing KHSRP were treated with CHX (100 μg/ml) for the indicated time points. The cell lysates were examined by Western blot

To elucidate the molecular mechanism by which KHSRP promotes metastasis in NSCLC cells, we next sought to identify proteins that interact with KHSRP in NSCLC cells. We performed tandem affinity purification (TAP) followed by mass spectrometry using Flag- and HA-tagged-KHSRP or control in HEK-293 T cells (Fig. 4a). Mass spectrometry identified 20 potential KHSRP-interacting proteins based on the criteria of unique peptides >2 and *p* < 0.05 with triple repeat appearances (Fig. 4b, c). The CO-IP experiment showed that the interaction between HNRNPC and KHSRP could be observed by immunoprecipitation in HEK-293 T cells with overexpression of KHSRP. On the contrary, the interaction between KHSRP and HNRNPC could also be observed by immunoprecipitation in HEK-293 T cells with overexpression of HNRNPC. The Co-IP experiments demonstrated that KHSRP and HNRNPC could coprecipitate with each other in HEK-293 T cells (Fig. 4d). Furthermore, the Co-IP experiments using endogenous proteins also demonstrated that KHSRP and HNRNPC could interact with each other (Fig. 4e). The immunofluorescence staining results showed that the KHSRP protein was colocalized and coexpressed with HNRNPC in the nucleus (Fig. 4f). Taken together, our results were consistent with the tandem affinity purification-mass spectrometry (TAP-MS) results. In order to further clarify the regulatory relationship between KHSRP and HNRNPC, we used Western blotting method to verify their expressions. Western blotting results showed that KHSRP could regulate the expression of HNRNPC, while HNRNPC could not regulate the expression of KHSRP, indicating that HNRNPC was a downstream protein of KHSRP (Fig. 4g). To verify whether KHSRP could stabilize the HNRNPC protein, we examined the effect of both KHSRP depletion and overexpression on the stability of endogenous HNRNPC protein in the presence of the protein synthesis inhibitor cycloheximide (CHX). The half-life of the KHSRP protein was not significantly different in A549 KHSRP knockdown cells and NCI-H292 KHSRP overexpression cells compared to that in the control cells (Fig. 4h). These data collectively suggest that KHSRP could specifically interact with HNRNPC to form a complex in the nucleus of NSCLC cells.

### HNRNPC promoted NSCLC cell proliferation, migration and invasion

**Fig. 5:**
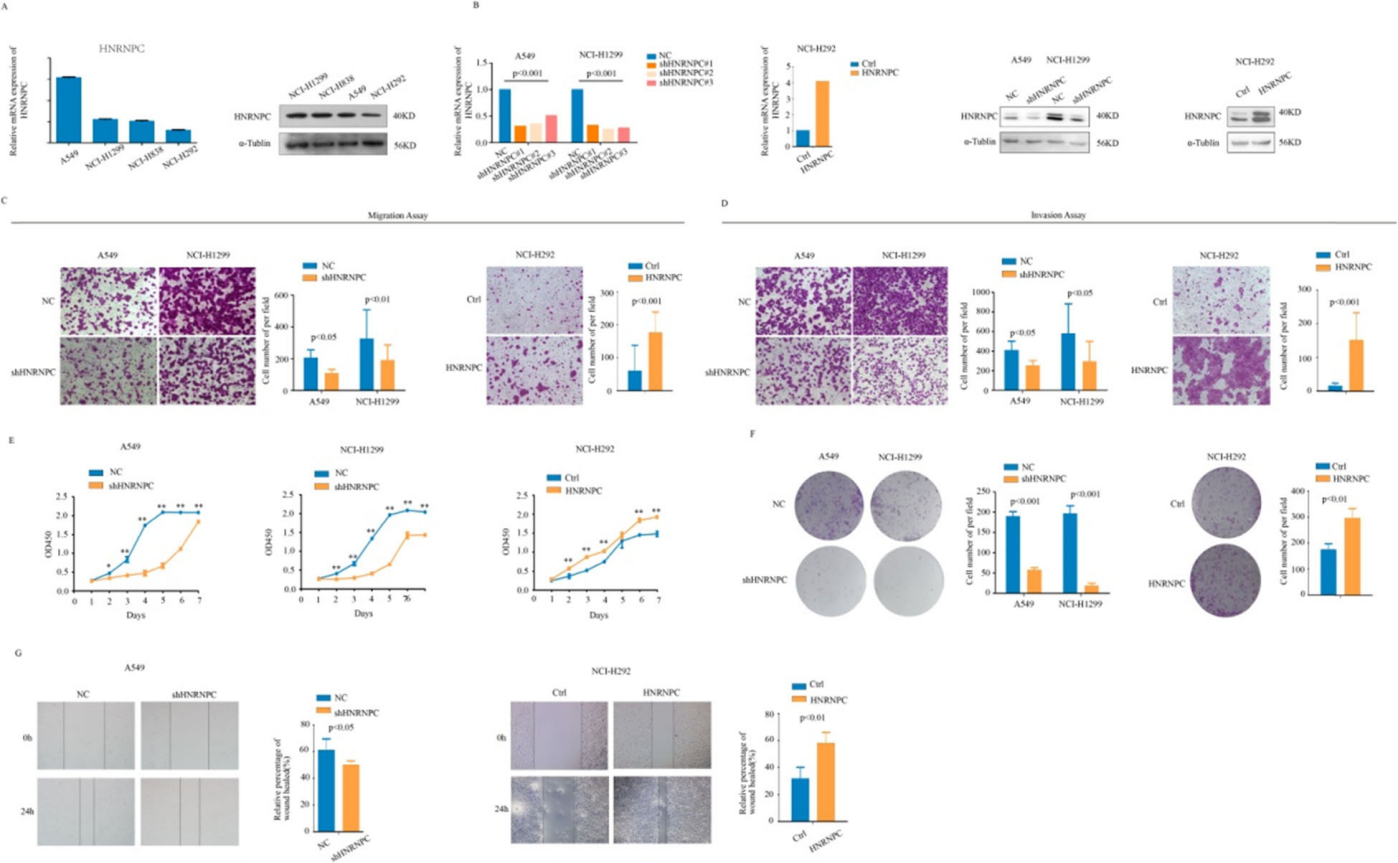
HNRNPC promoted NSCLC cell proliferation, migration and invasion. **a** The levels of HNRNPC expression in 4 human NSCLC cell lines were measured by real-time PCR and Western blot. **b** The mRNA and protein levels of HNRNPC in HNRNPC knockdown and negative control A549 cells and in HNRNPC overexpression and negative control NCI-H1299 cells were determined by real-time PCR and Western blotting analyses, respectively. **c** The effect of KHSRP on cell proliferation was evaluated by a CCK-8 assay in A549, NCI-H1299 and NCI-H292 cells. **d** The role of KHSRP in cell proliferation was evaluated by clone formation assays in A549, NCI-H1299 and NCI-H292 cells. **e** The effect of HNRNPC on cell migration was assessed by Transwell migration assays in A549, NCI-H1299 and NCI-H292 cells. **f** The effect of HNRNPC on cell invasion was assessed by Matrigel invasion assays in A549, NCI-H1299 and NCI-H292 cells. **g** Wound-healing assays were performed in A549, NCI-H1299 and NCI-H292 cells

First, we detected the expression of HNRNPC in 4 cell lines using real-time PCR and Western blotting. The expression trend of HNRNPC was consistent with that of KHSRP (Fig. 5a). To investigate the functional roles of HNRNPC in lung cancer progression, the transient knockdown of HNRNPC using siRNAs was established, and the interference efficiencies of the three interference fragments were over 50%. Then, we established stable models of HNRNPC knockdown in the A549 and NCI-H1299 cell lines as well as stable models of HNRNPC overexpression in the NCI-H292 cell line (Fig. 5b). CCK-8 and clone formation assays showed that HNRNPC knockdown substantially reduced the proliferation ability of A549 and NCI-H1299 cells, whereas the overexpression of HNRNPC significantly enhanced the proliferation ability of NCI-H292 cells (Fig. 5c, d). Moreover, the migration and Matrigel invasion assays showed that the knockdown of HNRNPC in A549 and NCI-H1299 cells significantly inhibited the cell migration (*P* < 0.05, Fig. 5e) and invasion potential (*P* < 0.05, Fig. 5f), whereas the overexpression of KHSRP in NCI-H292 cells markedly increased the cell migration (*P* < 0.001, Fig. 5e) and invasion potential (*P* < 0.001, Fig. 5f). Similarly, wound-healing assays showed the same role for HNRNPC as that shown by the migration and Matrigel invasion assays (*P* < 0.05, Fig. 5g). Overall, the knockdown of HNRNPC significantly inhibited the proliferation, migration, and invasion of NSCLC cells, whereas the overexpression of HNRNPC markedly promoted the proliferation, migration, and invasion of NSCLC cells in vitro.

### KHSRP induced human lung cancer cell invasion and metastasis by activating the IFN-α-JAK-p-STAT1 signaling pathway

**Fig. 6:**
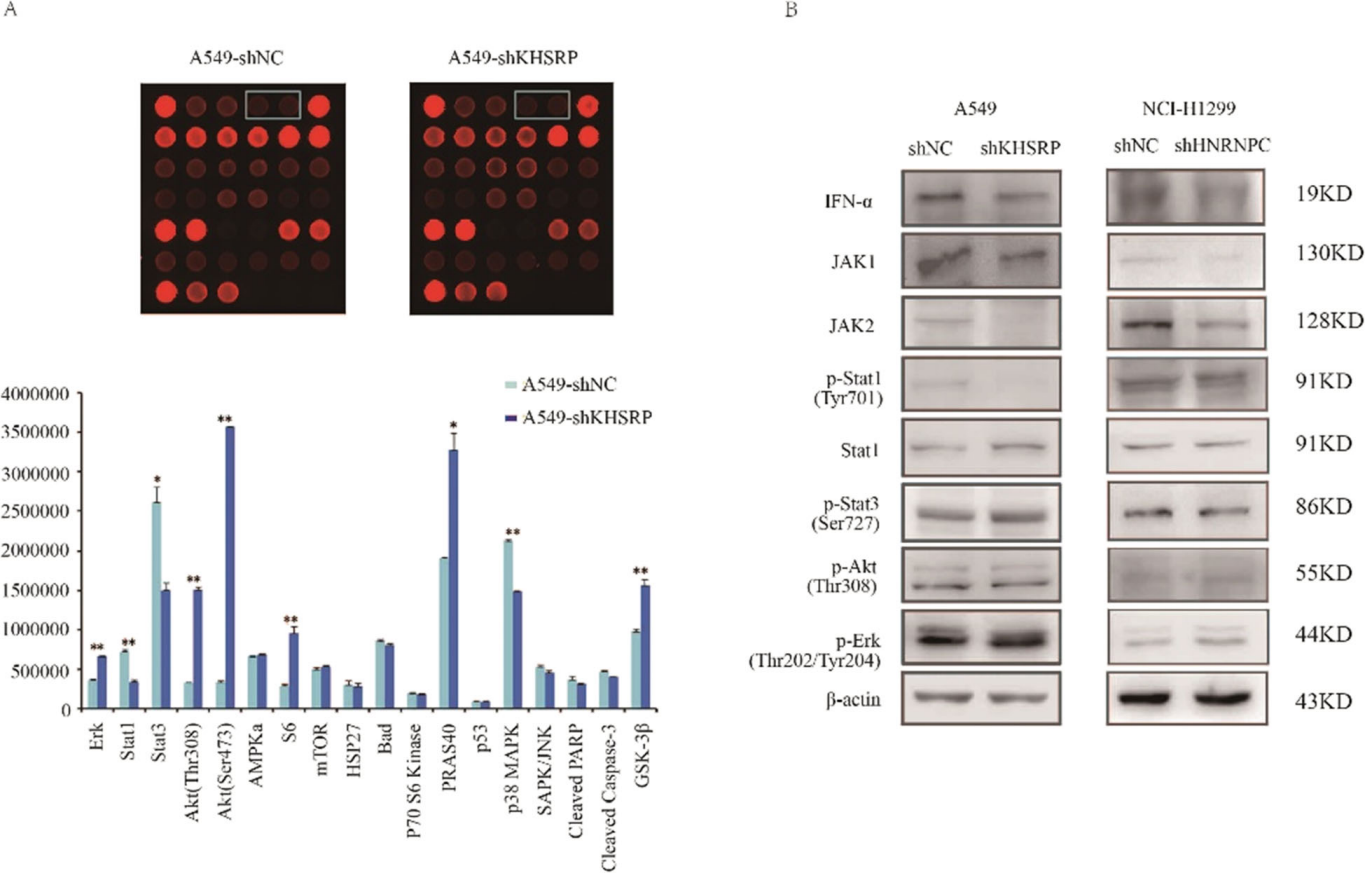
KHSRP induced human lung cancer cell invasion and metastasis by activating the JAK-STAT1 signaling pathway. **a** A human protein array was used to compare the differential expression of key molecules in tumor-related signaling pathways. **b** Western blot analysis was used to validate the expression differences in A549 cells upon the knockdown of KHSRP

Currently, the identities of KHSRP-associated signaling molecules that are responsible for mediating human lung cancer cell metastasis are unclear. To further elucidate the molecular mechanism of KHSRP in regulating NSCLC metastasis, a human protein array was utilized to compare the relative levels of 18 key molecules related to tumor signaling pathways between A549 cells with or without KHSRP knockdown. The intensities of the spots on the array were quantified by Image J analysis. Spots with statistical significance were selected to further validate the expression differences by Western blotting. The results show that 9 key molecules were altered, including ERK1/2, STAT1, STAT3, Akt (Thr308), Akt (Ser473), S6, PRAS40, p38 MAPK and GSK3β (Fig. 6a), of which only STAT1 could be validated in A549 cells. The expression of key molecules in signaling pathway was detected by knockdown of KHSRP in A549 cells and knockdown of HNRNPC in NCI-H1299 cells. The results showed that the key molecules of IFN-α, JAK1, JAK2, p-STAT1 were changed obviously, but p-STAT3, p-Akt, p-Erk had no change. The results indicated that KHSRP could interact with HNRNPC and HNRNPC may promote the metastasis of lung cancer through IFN-α-JAK-p-STAT1 signaling pathway (Fig. 6b).

### KHSRP expression parallels that of HNRNPC and is correlated with poor prognosis in NSCLC

**Fig. 7:**
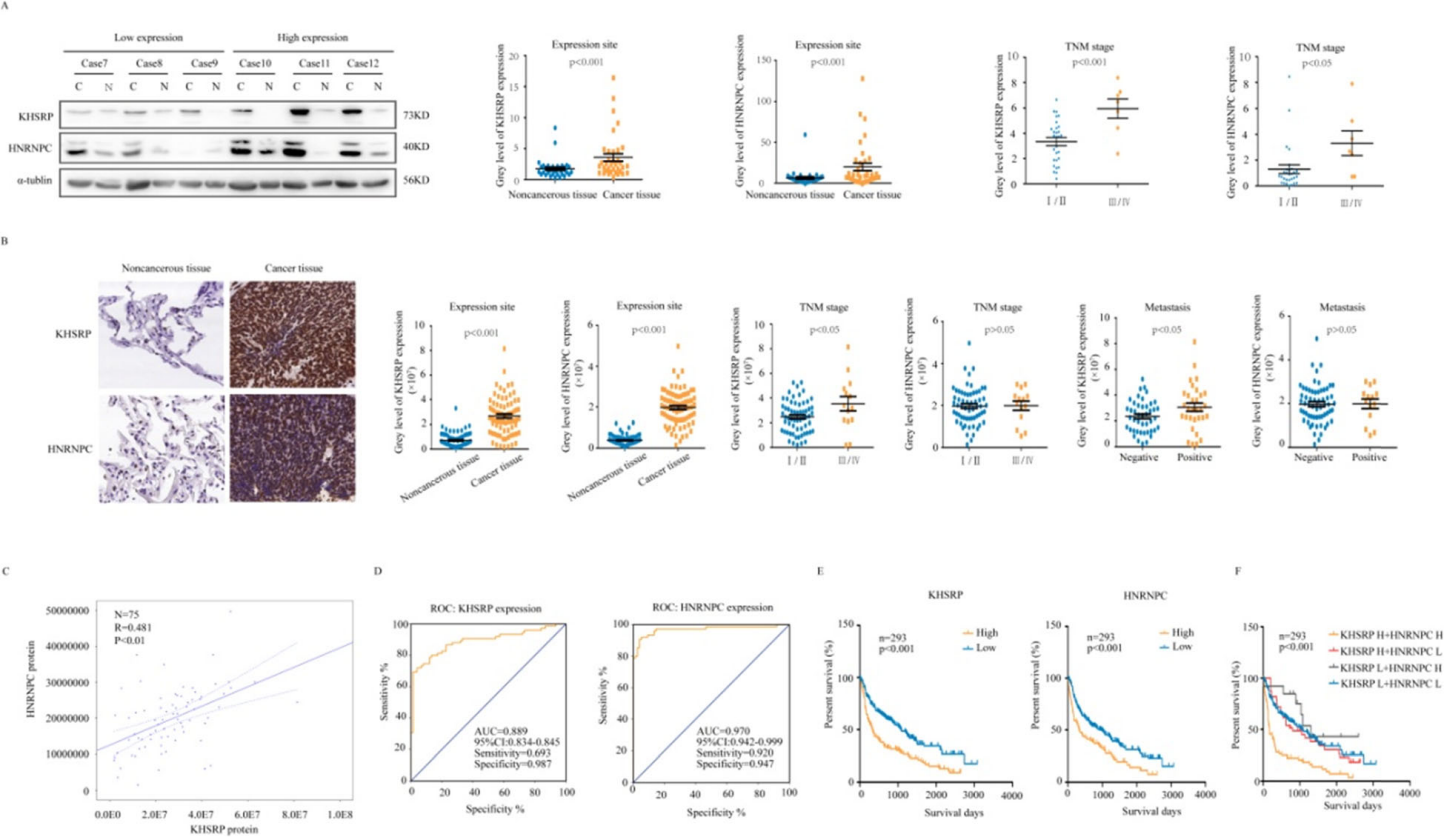
KHSRP expression paralleled that of HNRNPC and correlated with the poor prognosis of NSCLC patients. **a** Representative Western blot of KHSRP and HNRNPC in lung cancer tissues and their corresponding adjacent noncancerous tissues. The association between KHSRP/HNRNPC expression and the TNM stage in patients with lung cancer is shown. **b** Representative immunohistochemical staining of KHSRP and HNRNPC in lung cancer tissues and their corresponding adjacent noncancerous tissues. The association between KHSRP/HNRNPC expression and TNM stage/lymphatic metastasis in patients with lung cancer is shown. **c** The correlations between the protein levels of KHSRP and HNRNPC are shown. **d** The receiver operating characteristic (ROC) curves for predicting patient survival time using KHSRP or HNRNPC expression. **e** Kaplan–Meier analysis of overall survival according to KHSRP or HNRNPC expression levels. **f** Kaplan–Meier analysis of overall survival according to the combination of the above two indices

To determine whether the expression of KHSRP and HNRNPC in NSCLC is related to the prognosis of patients, we performed Western blotting analysis of 36 pairs of cancerous and noncancerous fresh tissues from NSCLC patients. The expression levels of KHSRP and HNRNPC in the 36 NSCLC tissue specimens were higher than those in adjacent noncancerous tissues (Additional file 1: Figure S4). Similar results were observed from the TCGA database (Additional file 1: Figure S5A). Moreover, high KHSRP or HNRNPC protein expression was associated with an advanced TNM stage (Fig. 7a). However, we detected the expression of KHSRP in 73 pairs of NSCLC and adjacent noncancerous lung tissues using real-time PCR. The mRNA expression levels of KHSRP in the 73 NSCLC tissue specimens were higher than those in adjacent noncancerous tissues, but there was not significantly associated with the TNM stage, lymph node and distant site metastasis (*P* > 0.05) (data not shown). In addition, the Oncomine database (www.oncomine.com) showed that KHSRP and HNRNPC were increased in particular types of solid tumors, including breast, colorectal and sarcoma (Additional file 1: Figure S5B). UALCAN (http://ualcan.path.uab.edu/) showed different expression patterns of KHSRP and HNRNPC in a variety of tumors (Additional file 1: Figure S5C). Then, we performed immunohistochemistry analysis of 75 pairs of cancerous and noncancerous tissues from NSCLC patients. Statistically, the overall expression of KHSRP and HNRNPC was much higher in cancerous tissues than in the adjacent noncancerous tissues (*P* < 0.001). In 75 NSCLC specimens, high expression of KHSRP was found in 52 cases of NSCLC, and low expression of KHSRP was found in 23 cases of NSCLC; high expression of HNRNPC was found in 57 cases of NSCLC, and low expression of HNRNPC was found in 18 cases of NSCLC. The protein expression level of KHSRP was significantly associated with the TNM stage (*P* < 0.05) and lymph node and distant site metastasis (*P* < 0.05) but was not associated with sex, age or tumor size (Table 1). There was no correlation between HNRNPC expression and tumor stage or metastasis (*P* > 0.05) (Table 2, Fig. 7b, Additional file 1: Figure S6). Importantly, KHSRP protein expression was positively correlated with that of HNRNPC, suggesting a potential KHSRP-HNRNPC pathway in lung cancer tissues (*R* = 0.481, *P* < 0.01) (Fig. 7c). The ROC curves illustrated that the areas under the curve of the KHSRP- and HNRNPC-based predictions were 0.889 and 0.970, respectively, suggesting that they could both potentially be applied for the prediction of patient survival (Fig. 7d). To further explore the role of KHSRP and HNRNPC in predicting cancer prognosis, we analyzed the KHSRP and HNRNPC mRNA expression and the corresponding clinical data from the publicly available GEO database (GSE30219, *n* = 293; GSE102287, *n* = 34). Kaplan-Meier survival analysis showed that higher expression levels of both KHSRP and HNRNPC were strongly associated with a shorter survival time for lung cancer patients (Fig. 7e, Additional file 1: Figure S7A). Interestingly, the shortest survival time was observed in the group with the highest expression of both KHSRP and HNRNPC (Fig. 7f). In addition, the Kaplan-Meier Plotter database (www.kmplot.com) showed that the Kaplan-Meier survival analysis result was the same as the above result (Additional file 1: Figure S7B). Taken together, these findings indicate that KHSRP plays a critical role in lung cancer development and metastasis and might be a potential prognostic biomarker for this disease.

**Table 1:** Correlation between KHSRP levels in NSCLC patients and their clinicopathologic characteristics

Clinical pathology	KHSRP Levels			N	*P* Value
	Low	Middle	High		
Gender					
Male	5 (12.82%)	15 (38.46%)	19 (48.72%)	39	
Female	10 (27.78%)	10 (27.78%)	16 (44.44%)	36	0.2338
Age					
< 60	6 (20.00%)	6 (20.00%)	18 (60.00%)	30	
≥ 60	9 (20.00%)	19 (42.22%)	17 (37.78%)	45	0.2730
Tumor size (cm)					
≤ 3	7 (17.07%)	13 (31.71%)	21 (51.22%)	41	
> 3	8 (23.53%)	12 (35.29%)	14 (41.18%)	34	0.4663
Histological grade					
I- II	2 (15.38%)	5 (38.46%)	6 (46.15%)	13	
II	10 (22.73%)	15 (34.09%)	19 (43.18%)	44	0.3785
II - IV	3 (16.67%)	5 (27.78%)	10 (55.56%)	18	
Clinical Stage					
T1 + T2	13 (5.45%)	20 (36.36%)	22 (40.00%)	55	
T3 + T4	2 (10.00%)	5 (25.00%)	13 (65.00%)	20	0.0121*
Lymph node status					
Metastasis	2 (9.52%)	6 (28.57%)	13 (61.90%)	21	
No metastasis	13 (24.07%)	19 (35.19%)	22 (40.74%)	54	0.0166*
Carcinoma					
Primary	15 (20.00%)	25 (33.33%)	35 (46.67%)	75	
Adjacent	60 (80.00%)	12 (16.00%)	3 (4.00%)	75	0.0000*

*P* value represents the probability from a chi-square test for tissue KHSRP levels between variable subgroups, *P < 0.05

**Table 2:** Correlation between HNRNPC levels in NSCLC patients and their clinicopathologic characteristics

Clinical pathology	HNRNPC levels			N	*P* Value
	Low	Middle	High		
Gender					
Male	5 (12.82%)	15 (38.46%)	19 (48.72%)	39	
Female	10 (27.78%)	10 (27.78%)	16 (44.44%)	36	0.8181
Age					
< 60	6 (20.00%)	6 (20.00%)	18 (60.00%)	30	
≥ 60	9 (20.00%)	19 (42.22%)	17 (37.78%)	45	0.4617
Tumor size (cm)					
≤ 3	7 (17.07%)	13 (31.71%)	21 (51.22%)	41	
> 3	8 (23.53%)	12 (35.29%)	14 (41.18%)	34	0.6676
Histological grade					
I- II	2 (15.38%)	5 (38.46%)	6 (46.15%)	13	
II	10 (22.73%)	15 (34.09%)	19 (43.18%)	44	0.1887
II - IV	3 (16.67%)	5 (27.78%)	10 (55.56%)	18	
Clinical Stage					
T1 + T2	13 (5.45%)	20 (36.36%)	22 (40.00%)	55	
T3 + T4	2 (10.00%)	5 (25.00%)	13 (65.00%)	20	0.7696
Lymph node status					
Metastasis	2 (9.52%)	6 (28.57%)	13 (61.90%)	21	
No metastasis	13 (24.07%)	19 (35.19%)	22 (40.74%)	54	0.4323
Carcinoma					
Primary	15 (20.00%)	25 (33.33%)	35 (46.67%)	75	
Adjacent	60 (80.00%)	12 (16.00%)	3 (4.00%)	75	0.0000*

*P* value represents the probability from a chi-square test for tissue HNRNPC levels between variable subgroups, *P < 0.05

## Discussion

KHSRP is a multifunctional nucleic acid-binding protein belonging to the far upstream component binding protein (FUBPs) family, a single-stranded DNA-binding family that includes three members, FUBPl, FUBP2, and FUBP3 [12]. The gene is located on chromosome 19p13.3 and contains 711 amino acids. Its structure is composed of three parts: the terminal domain of the amino acid, the central domain containing four KH motifs and the terminal domain of the carboxyl group [13]. The three highly conserved domains are linked by variable joining regions. There are four tyrosine-rich motifs in the carboxyl terminal domain. The central KH domain is the most characteristic domain. In addition to its direct binding with ribonucleic acid, there is a β-fold structure outside of the KH1 and KH4 domains that can interact with other proteins, and negative regulatory binding sites exist in the KH2 and KH3 domains. KHSRP can also interact with other proteins [14]. Phosphorylation of the KH1 domain opens its folding to establish a new protein-binding site. KHSRP binds not only ribonucleic acid but also other proteins, which can interact with other proteins and play a regulatory role [12]. KHSRP plays an important role in regulating RNA splicing, RNA transport, RNA editing, and mRNA stabilization and degradation [15]. It is involved in the regulation of neuromuscular disorders [16], obesity [17], type II diabetes [18], cancer [19] and other cellular processes [20, 21].

To date, several studies have focused on the role of KHSRP in tumorigenesis and development. It seems that KHSRP plays different roles in the metastasis and invasion of distinct cancers. Jian Yang et al. [22] reported that KHSRP does not affect the proliferation of human glioma cells, but low expression of KHSRP can promote the formation of tumors. He Li et al. [23] reported that low expression of KHSRP can promote the formation of gastric cancer and is related to the prognosis of gastric cancer patients. However, some studies have found that KHSRP plays an opposite role in liver cancer. For example, Mona Malz et al. [24] found that FUBP1 and FUBP2 can promote the proliferation and invasion of liver cancer, and Ramdzan M et al. [25] found that FUBP1 and FUBP2 are highly expressed in liver cancer tissues with medium and low levels of differentiation. Gagne JP et al. [26] detected the FUBPl and FUBP2 proteins in a study on human ovarian cancer cells, but they did not further study or analyze the association between ovarian cancer and FUBPl and FUBP2. To date, there have been few reports describing the role of these proteins in the metastasis of lung cancer [27]. Regarding the function and role of KHSRP in lung cancer, the research results of different scholars have not been consistent. Chien MH et al. [28] reported that KHSRP is correlated with a long survival rate of NSCLC patients and inhibits the in vitro mobility and in vivo metastasis of NSCLC cells, suggesting a tumor-suppressive role for KHSRP in lung cancer. In contrast, Bikkavilli RK et al. [29] reported that silencing KHSRP decreased cell proliferation, reversed anchorage-independent growth, and reduced migration/invasion, suggesting an oncogenic role for KHSRP in lung cancer. In our study, a series of in vitro and in vivo assays were conducted to clarify the biological functions of KHSRP in regulating NSCLC cell invasion and metastasis. We found that interference with KHSRP could inhibit the migration and invasion of lung cancer cells in vitro and inhibit the growth and metastasis of lung cancer cells in vivo. Higher expression of KHSRP was observed in lung cancer tissues compared to that in adjacent noncancerous tissues. Increased KHSRP expression was significantly associated with advanced tumor stages as well as lymph node and distant metastasis. These results indicate that KHSRP is a potential prognostic marker and therapeutic molecular target in lung cancer.

HNRNPC is an RNA-binding protein located in the nucleus that belongs to the subfamily of ubiquitously expressed heterogeneous nuclear ribonucleoproteins (hnRNPs). HNRNPC is associated with pre-mRNAs in the nucleus and plays multiple roles in posttranscriptional regulation, including roles in alternative splicing [30, 31], nuclear retention and export [32], stability [33] and translation [34]. HNRNPC is thought to be a prognostic marker in tumors [35] and has been demonstrated to be highly expressed in multiple tumors, including hepatocellular carcinoma [36], breast cancer [37], glioblastoma [38], and ovarian cancer [39]. However, the exact function and molecular mechanism of HNRNPC in lung cancer are still unclear. In this study, we demonstrated that KHSRP colocalized and coimmunoprecipitated with HNRNPC in lung cancer cells, suggesting that KHSRP can specifically interact with HNRNPC to form a complex in the nuclei of NSCLC cells. We also showed that knockdown of HNRNPC significantly inhibited the proliferation, migration, and invasion of NSCLC cells, whereas overexpression of HNRNPC markedly promoted the proliferation, migration, and invasion of NSCLC cells in vitro, suggesting that HNRNPC has an indispensable role in lung cancer. It is well recognized that the prognosis of patients with lung cancer is very poor due to distant metastasis. To gain insight into the correlation of HNRNPC expression and the prognosis of NSCLC patients, we used Western blot to demonstrate that HNRNPC protein expression was high in cancerous tissues and associated with advanced TNM stages, but no correlation with tumor stage or metastasis was observed by immunohistochemistry analysis. Kaplan-Meier survival analysis showed that high expression of HNRNPC was strongly associated with a short survival time for lung cancer patients. Moreover, the shortest survival time was observed in the group with high expression of both KHSRP and HNRNPC. Based on the above results, we speculate that HNRNPC plays a critical role in lung cancer development and metastasis.

To date, however, the molecular mechanisms by which KHSRP promotes lung cancer cell migration and invasion have not been elucidated. Previous studies have reported that activation of the KHSRP-mediated nuclear factor-κB, PI3K/AKT or p38 signaling pathway is closely associated with lung tumorigenesis and metastasis [16, 40, 41]. However, the detailed regulatory mechanisms remain unclear. In this study, the expression levels of IFN-α, JAK1, JAK2 and p-STAT1 were decreased by silencing KHSRP or HNRNPC. We elucidated that KHSRP could interact with HNRNPC and HNRNPC-mediated tumor growth and metastasis could be, at least partly, attributed to the activation of IFN-α-JAK-p-STAT1 signaling pathway, which is critical for tumorigenesis and metastasis in NSCLC. Therefore, the IFN-α-JAK-p-STAT1 signaling pathway might be responsible for the oncogenic function of KHSRP in NSCLC. Further work is needed to clarify the mechanisms by which HNRNPC activates the JAK-STAT1 signaling pathway in detail.

## Conclusions

**Fig. 8:**
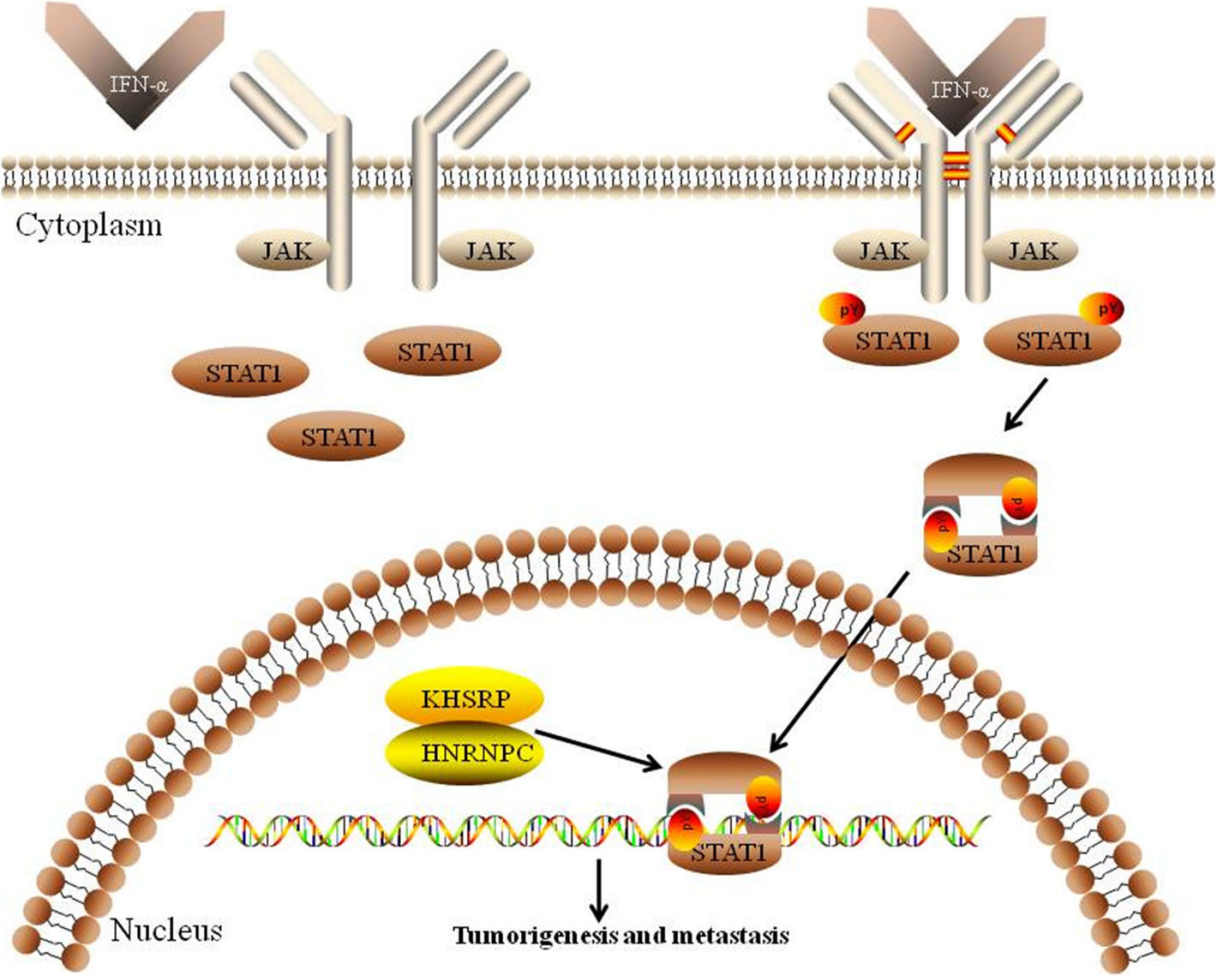
Schematic model of the potential mechanism of KHSRP regulation in the metastasis of lung cancer. In this proposed model, KHSRP can interact with HNRNPC and form a complex, activating the JAK-STAT1 signaling pathway and thus leading to the invasion and metastasis of lung cancer cells

In summary, the present study demonstrates that KHSRP and HNRNPC play important roles in the development and progression of NSCLC. Meanwhile, KHSRP and HNRNPC can interact with each other and activate the IFN-α-JAK-p-STAT1 signaling pathway, which ultimately increases the invasion and metastasis of lung cancer cells (Fig. 8). More importantly, our investigation reveals that high expression levels of KHSRP and HNRNPC are significantly correlated with tumor metastasis and serve as independent prognostic factors for the poor outcomes of NSCLC patients. Altogether, the present study results suggest that KHSRP may serve as a promising therapeutic target for the prevention and treatment of NSCLC invasion and metastasis.

## Supplementary information

**Additional file 1: Figure S1.** Bioinformatics was used to analyze the cellular components, molecular functions and biological processes. **Figure S2.** Nuclear proteins were verified by qRT-PCR and western blot analysis. **Figure S3.** The *in vitro* migration and invasion abilities of cells transfected with siRNAs of KHSRP, PSIP1 and VASP were evaluated. **Figure S4.** Thirty-six pairs of cancerous and noncancerous fresh tissues from NSCLC patients were analyzed by Western blot. **Figure S5.** The expression of KHSRP and HNRNPC in various network databases. **Figure S6.** A total of 75 pairs of cancerous and noncancerous fresh tissues from NSCLC patients were analyzed by immunohistochemistry analysis. **Figure S7.** Kaplan-Meier survival analysis was performed to explore the roles of KHSRP and HNRNPC in predicting cancer prognosis. **Table S1.** Primer sequences for real-time PCR used in the study. **Table S2.** Primer sequences for siRNA used in the study. **Table S3.** The 52 up-regulated differential expression proteins identified by iTraq and SWATH^TM^two proteomics methods. **Table S4.** The 64 down-regulated differential expression proteins identified by iTraq and SWATH^TM^two proteomics methods.

## Data Availability

All data generated or analyzed during this study are included either in this article or in the supplementary information files.

## Abbreviations

ATCC: American Type Culture Collection
CCK-8: Cell Counting Kit-8
Co-IP: Coimmunoprecipitation
DMEM: Dulbecco’s Modified Eagle’s Medium
FBS: Fetal bovine serum
H & E: Hematoxylin and eosin
HNRNPC: Heterogeneous nuclear ribonucleoprotein C (C1/C2)
IHC: Immunohistochemistry
ITRAQ: Isobaric tags for relative and absolute quantitation
KHSRP: KH-type splicing regulatory protein; far upstream element-binding protein 2, KHSRP/KSRP/FBP2/FUBP2
KM: Kaplan-Meier
Nano-LC-MS/MS: Nanoliquid chromatography-tandem mass spectrometry
NSCLC: Non-small cell lung cancer
PVDF: Polyvinylidene fluoride
qPCR: Real-time polymerase chain reaction
RPMI 1640: Roswell Park Memorial Institute 1640
siRNA: Small-interfering RNA
SPF: Specific pathogen-free
TAP: Tandem affinity purification
WHO: World Health Organization

## References

[1] Bray F, Ferlay J, Soerjomataram I, Siegel RL, Torre LA, Jemal A (2018). Global cancer statistics 2018: GLOBOCAN estimates of incidence and mortality worldwide for 36 cancers in 185 countries. CA Cancer J Clin.

[2] Ferlay J, Colombet M, Soerjomataram I, Mathers C, Parkin DM, Piñeros M (2019). Estimating the global cancer incidence and mortality in 2018: GLOBOCAN sources and methods. Int J Cancer.

[3] Heist RS, Engelman JA (2012). SnapShot: non-small cell lung cancer. Cancer Cell.

[4] Parguiña AF, Rosa I, García A (2012). Proteomics applied to the study of platelet-related diseases: aiding the discovery ofnovel platelet biomarkers and drug targets. J Proteome.

[5] Tan HT, Lee YH, Chung MCM (2012). Cancer proteomics. Mass Spectrom Rev.

[6] Indovina P, Marcelli E, Pentimalli F, Tanganelli P, Tarro G, Giordano A (2013). Massspectrometry-based proteomics: the road to lung cancer biomarker discovery. Mass Spectrom Rev.

[7] Lin HC, Zhang FL, Geng Q, Yu T, Cui YQ, Liu XH (2013). Quantitative proteomic analysis identifies CPNE3 as a novel metastasis-promoting gene in NSCLC. J Proteome Res.

[8] Zhang F, Lin H, Aiqin G, Li J, Liu L, Yu T (2014). SWATHTM- and iTRAQ-based quantitative proteomic analyses reveal an overexpression and biological relevance of CD109 in advanced NSCLC. J Proteome.

[9] Kuang X-Y, Jiang H-S, Li K, Zheng Y-Z, Liu Y-R, Qiao F (2016). The phosphorylation-specific association of STMN1 with GRP78 promotes breast cancer metastasis. Cancer Lett.

[10] Li J, Cheng D, Zhu M, Yu H, Pan Z, Liu L (2019). OTUB2 stabilizes U2AF2 to promote the Warburg effect and tumorigenesis via the AKT/mTOR signaling pathway in non-small cell lung cancer. Theranostics.

[11] Yu T, Li J, Yan M, Liu L, Lin H, Zhao F (2015). MicroRNA-193a-3p and -5p suppress the metastasis of human non-small-cell lung cancer by downregulating the ERBB4/PIK3R3/mTOR/S6K2 signaling pathway. Oncogene.

[12] Gherzi R, Chen CY, Trabucchi M, Ramos A, Briata P (2010). The role of KSRP in mRNA decay and microRNA precursor maturation. Wiley Interdiscip Rev RNA.

[13] Nicastro G, García-Mayoral MF, Hollingworth D, Kelly G, Martin SR, Briata P (2012). Noncanonical G recognition mediates KSRP regulation of let-7 biogenesis. Nat Struct Mol Biol.

[14] Díaz-Moreno I, Hollingworth D, Frenkiel TA, Kelly G, Martin S, Howell S (2009). Phosphorylation-mediated unfolding of a KH domain regulates KSRP localization via 14-3-3binding. Nat Struct Mol Biol.

[15] Gherzi R, Lee K-Y, Briata P, Wegmuller D, Moroni C, Karin M (2004). A KH domain RNA binding protein, KSRP, promotes ARE-directed mRNA turnover by recruiting the degradation machinery. Mol Cell.

[16] Amirouche A, Tadesse H, Lunde JA, Bélanger G, Côté J, Jasmin BJ (2013). Activation of p38 signaling increases utrophin a expression in skeletal muscle via the RNA-binding protein KSRP and inhibition of AU-rich element-mediated mRNA decay: implications for novel DMD therapeutics. Hum Mol Genet.

[17] Lin YY, Chou CF, Giovarelli M, Briata P, Gherzi R, Chen CY (2014). KSRP and MicroRNA 145 are negative regulators of lipolysis in white adipose tissue. Mol Cell Biol.

[18] Briata P, Bordo D, Puppo M, Gorlero F, Rossi M, Bizzozzero NP (2016). Diverse roles of the nucleic acid binding protein KHSRP in cell differentiation and disease. Wiley Interdiscip Rev RNA.

[19] Yuan H, Deng R, Zhao X, Chen R, Hou G, Zhang H (2017). SUMO1 modification of KHSRP regulates tumorigenesis by preventing the *TL-G-Rich* miRNA biogenesis. Mol Cancer.

[20] Trabucchi M, Briata P, Filipowicz W, Ramos A, Gherzi R, Rosenfeld MG (2010). KSRP promotes the maturation of a group of miRNA precursors. Adv Exp Med Biol.

[21] King PH, Chen CY (2014). Role of KSRP in control of type I interferon and cytokine expression. J Interf Cytokine Res.

[22] Yang J, Fan J, Li Y, Li F, Chen P, Fan Y (2013). Genome-wide RNAi screening identifies genes inhibiting the migration of glioblastoma cells. PLoS One.

[23] He L, Wang J, Xu H, Xing R, Pan Y, Li W (2013). Decreased fructose-1,6-bisphosphatase-2 expression promotes glycolysis and growth in gastric cancer cells. Mol Cancer.

[24] Malz M, Weber A, Singer S, Riehmer V, Bissinger M, Riener MO (2009). Overexpression of far upstream element binding proteins: a mechanism regulating proliferation and migration in liver cancer cells. Hepatology.

[25] Zubaidah RM, Tan GS, Tan SBE, Lim SG, Lin Q, Chung MCM (2008). 2-D DIGE profiling of hepatocellular carcinoma tissues identified isoforms of far upstream binding protein (FUBP) as novel candidates in liver carcinogenesis. Proteomics.

[26] Gagne JP, Gagne P, Hunter JM, Bonicalzi ME, Lemay JF, Kelly I (2005). Proteome profiling of human epithelial ovarian cancer cell line TOV-112D. Mol Cell Biochem.

[27] Tong L, Luo Y, Wei T, Guo L, Wang H, Zhu W (2016). KH-type splicing regulatory protein (KHSRP) contributes to tumorigenesis by promoting miR-26a maturation in small cell lung cancer. Mol Cell Biochem.

[28] Chien MH, Lee WJ, Yang YC, Li YL, Chen BR, Cheng TY (2017). KSRP suppresses cell invasion and metastasis through miR-23a-mediated EGR3 mRNA degradation in non-small cell lung cancer. BiochimBiophys Acta Gene Regul Mech.

[29] Bikkavilli RK, Zerayesus SA, Van Scoyk M, Wilson L, Wu PY, Baskaran A (2017). K-homology splicing regulatory protein (KSRP) promotes post-transcriptional destabilization of Spry4 transcripts in non-small cell lung cancer. J Biol Chem.

[30] Wen J, Toomer KH, Chen Z, Cai X (2015). Genome-wide analysis of alternative transcripts in human breast cancer. Breast Cancer Res Treat.

[31] Tremblay MP, Armero VE, Allaire A, Boudreault S, Martenon-Brodeur C, Durand M (2016). Global profiling of alternative RNA splicing events provides insights into molecular differences between various types of hepatocellular carcinoma. BMC Genomics.

[32] Nakielny S, Dreyfuss G (1996). The hnRNP C proteins contain a nuclear retention sequence that can override nuclear export signals. J Cell Biol.

[33] Shen Y, Liu S, Fan J, Jin Y, Tian B, Zheng X (2017). Nuclear retention of the lncRNA SNHG1 by doxorubicin attenuates hnRNPC-p53 protein interactions. EMBO Rep.

[34] Gruber AJ, Schmidt R, Gruber AR, Martin G, Ghosh S, Belmadani M (2016). A comprehensive analysis of 3’ end sequencing data sets reveals novel polyadenylation signals and the repressive role of heterogeneous ribonucleoprotein C on cleavage and polyadenylation. Genome Res.

[35] Huang H, Han Y, Zhang C, Wu J, Feng J, Qu L (2016). HNRNPC as a candidate biomarker for chemoresistance in gastric cancer. Tumour Biol.

[36] Sun D-q, Wang Y, Liu D-g (2013). Overexpression of hnRNPC2 induces multinucleation by repression of Aurora B in hepatocellular carcinoma cells. Oncol Lett.

[37] Wu Y, Zhao W, Liu Y, Tan X, Li X, Zou Q (2018). Function of HNRNPC in breast cancer cells by controlling the dsRNA-induced interferon response. EMBO J.

[38] Park YM, Hwang SJ, Masuda K, Choi K-M, Jeong M-R, Nam D-H (2012). Heterogeneous Nuclear Ribonucleoprotein C1/C2 Controls the Metastatic Potential of Glioblastoma by Regulating PDCD4. Mol Cell Biol.

[39] Kleemann M, Schneider H, Unger K, Sander P, Schneider EM, Fischer-Posovszky P (2018). MiR-744-5p inducing cell death by directly targeting HNRNPC and NFIX in ovarian cancer cells. Sci Rep.

[40] Nie Y, Cao M, Wu D, Li N, Peng J, Yi S (2018). KH-type splicing regulatory protein is regulated by nuclear factor-κB signaling to mediate innate immunity in Caco-2 cells infected by Salmonella enteritidis. Folia Microbiol (Praha).

[41] Wang YY, Gu XL, Wang C, Wang H, Ni QC, Zhang CH (2016). The far-upstream element-binding protein 2 is correlated with proliferation and doxorubicin resistance in human breast cancer cell lines. Tumour Biol.

