# Supplementary material for: RNA-binding protein KHSRP promotes tumor growth and metastasis in non-small cell lung cancer
Source: J Exp Clin Cancer Res. 2019 Nov 27;38:478. doi: 10.1186/s13046-019-1479-2 (PMC6882349; doi:10.1186/s13046-019-1479-2)
Supplement: Supplementary file 1 — Additional file 1: Figure S1. Bioinformatics was used to analyze the cellular components, molecular functions and biological processes. Figure S2. Nuclear proteins were verified by qRT-PCR and western blot analysis. Figure S3. The in vitro migration and invasion abilities of cells transfected with siRNAs of KHSRP, PSIP1 and VASP were evaluated. Figure S4. Thirty-six pairs of cancerous and noncancerous fresh tissues from NSCLC patients were analyzed by Western blot. Figure S5. The expression of KHSRP and HNRNPC in various network databases. Figure S6. A total of 75 pairs of cancerous and noncancerous fresh tissues from NSCLC patients were analyzed by immunohistochemistry analysis. Figure S7. Kaplan-Meier survival analysis was performed to explore the roles of KHSRP and HNRNPC in predicting cancer prognosis. Table S1. Primer sequences for real-time PCR used in the study. Table S2. Primer sequences for siRNA used in the study. Table S3. The 52 up-regulated differential expression proteins identified by iTraq and SWATHTMtwo proteomics methods. Table S4. The 64 down-regulated differential expression proteins identified by iTraq and SWATHTMtwo proteomics methods. [file 13046_2019_1479_MOESM1_ESM.doc]

**Figure Legends and Tables**

Figure S1. Bioinformatics was used to analyze the cellular components, molecular functions and biological processes. (A) The histogram shows the average ratio distribution of proteins quantified by the iTRAQ-labeling and SWATH™ approaches. (B) Gene Ontology analysis revealed the main cellular components of the differentially expressed nuclear proteins. (C) Gene Ontology analysis revealed the main molecular functions of the differentially expressed nuclear proteins. (D) Gene Ontology analysis revealed the main biological processes of the differentially expressed nuclear proteins.

Figure S2. Nuclear proteins were verified by qRT-PCR and western blot analysis. (A) The mRNA expression levels of 9 potential NSCLC metastasis-associated proteins were determined in NCI-H1299, A549, NCI-H358 and NCI-H292 cell lines by qRT-PCR. (B) The expression levels of 5 potential NSCLC metastasis-associated proteins were determined in NCI-H1299, A549, NCI-H358 and NCI-H292 cell lines by western blot. (C) Gray value displayed the protein expression of KHSRP, PSPI1, DDX21, PTRF and VASP proteins in NCI-H1299, A549, NCI-H358 and NCI-H292 cell lines.

Figure S3. The *in vitro* migration and invasion abilities of cells transfected with siRNAs of KHSRP, PSIP1 and VASP were evaluated. (A) The mRNA expression levels of KHSRP, PSIP1 and VASP were determined in A549 cell line by qRT-PCR. (B) The *in vitro* migration and invasion abilities of cells transfected with siRNAs of KHSRP, PSIP1 and VASP were evaluated using Transwell assays.

Figure S4. Thirty-six pairs of cancerous and noncancerous fresh tissues from NSCLC patients were analyzed by Western blot.

Figure S5. The expression of KHSRP and HNRNPC in various network databases. (A) The mRNA expression levels of KHSRP and HNRNPC were significantly increased in NSCLC tissues compared with those in noncancerous tissues according to the TCGA database. (B) The expression levels of KHSRP and HNRNPC were analyzed using the Oncomine database. (C) The different expression levels of KHSRP and HNRNPC were analyzed in a variety of tumors in the UALCAN database.

Figure S6. A total of 75 pairs of cancerous and noncancerous fresh tissues from NSCLC patients were analyzed by immunohistochemistry analysis.

Figure S7. Kaplan-Meier survival analysis was performed to explore the roles of KHSRP and HNRNPC in predicting cancer prognosis. (A) The KHSRP and HNRNPC mRNA expression and corresponding clinical data from the publicly available GEO database (GSE102287, n=34) were analyzed. (B) Kaplan-Meier survival analysis was performed using the Kaplan-Meier Plotter database.

Table S1. Primer sequences for real-time PCR used in the study

Table S2. Primer sequences for siRNA used in the study

Table S3. The 52 up-regulated differential expression proteins identified by iTraq and SWATH^TM^two proteomics methods

Table S4. The 64 down-regulated differential expression proteins identified by iTraq and SWATH^TM^two proteomics methods

Supplementary Figure 1


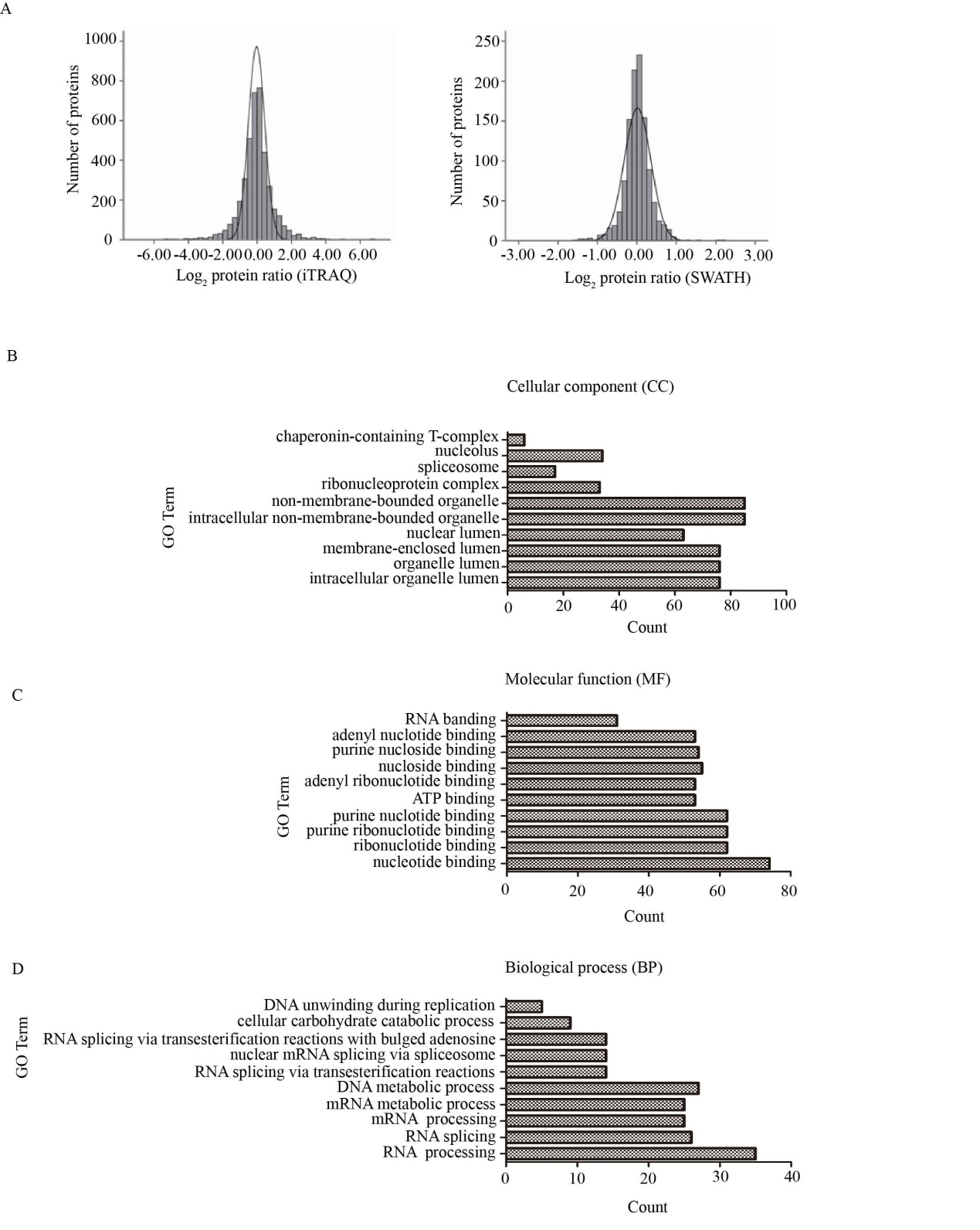


Supplementary figure 2


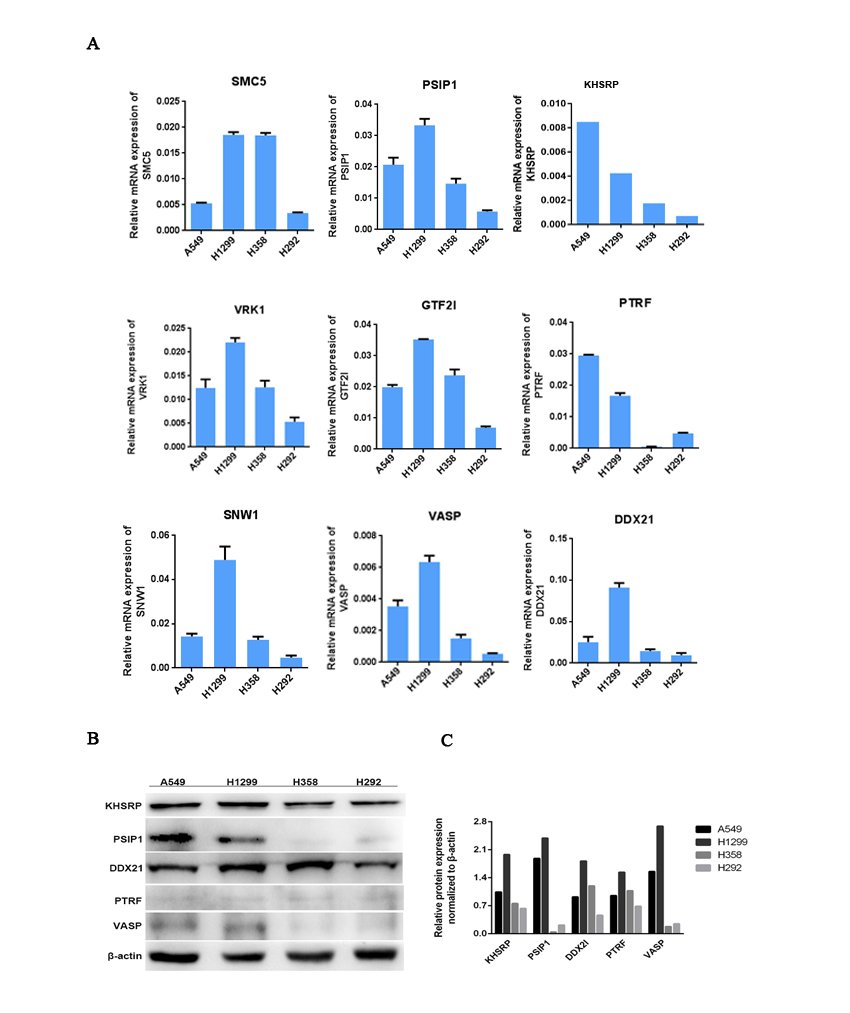


Supplementary figure 3


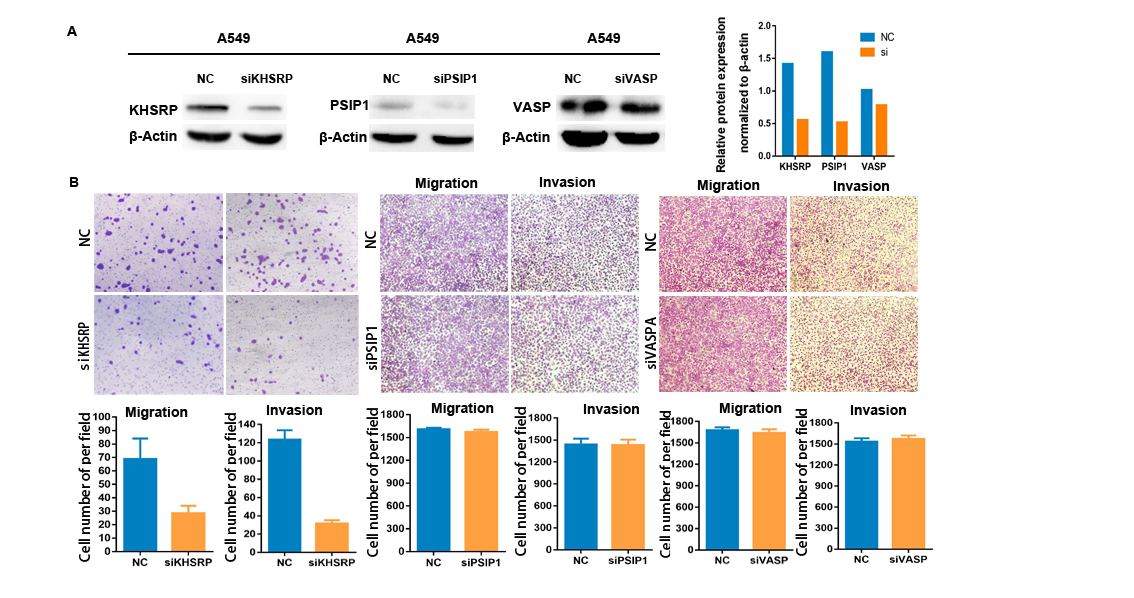


Supplementary figure 4


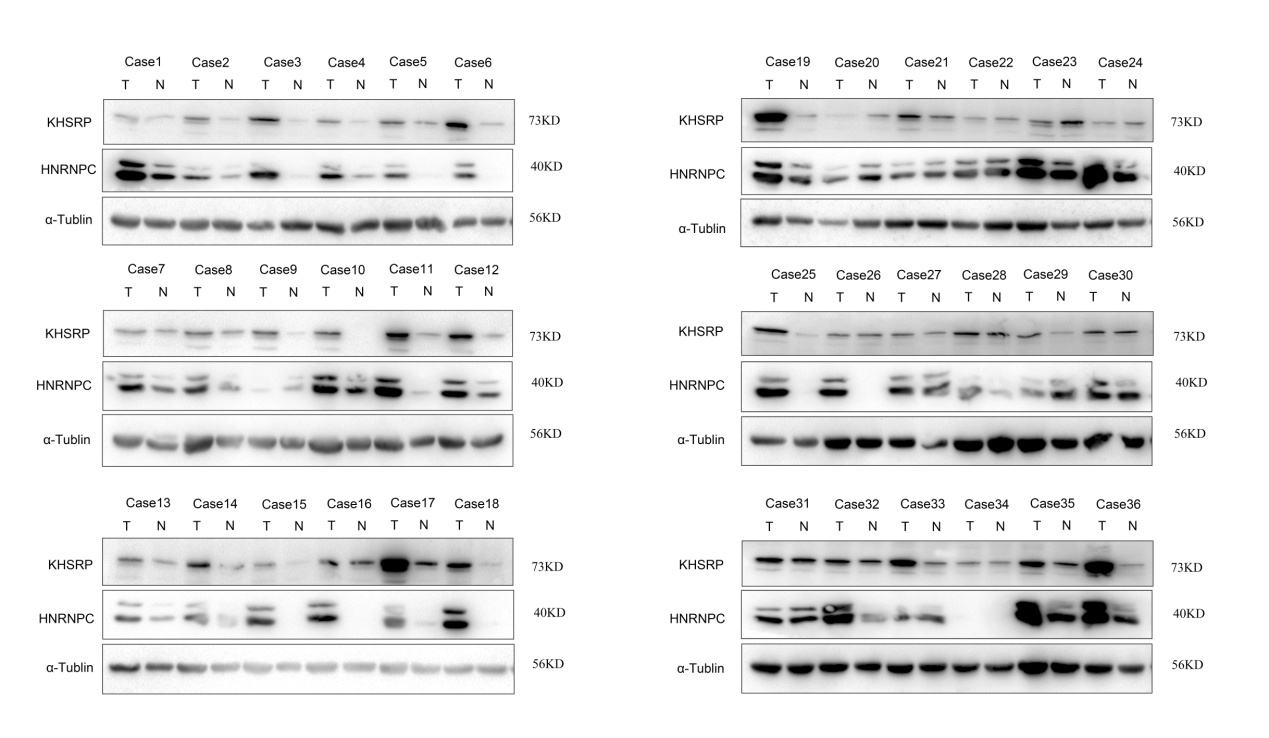


Supplementary figure 5


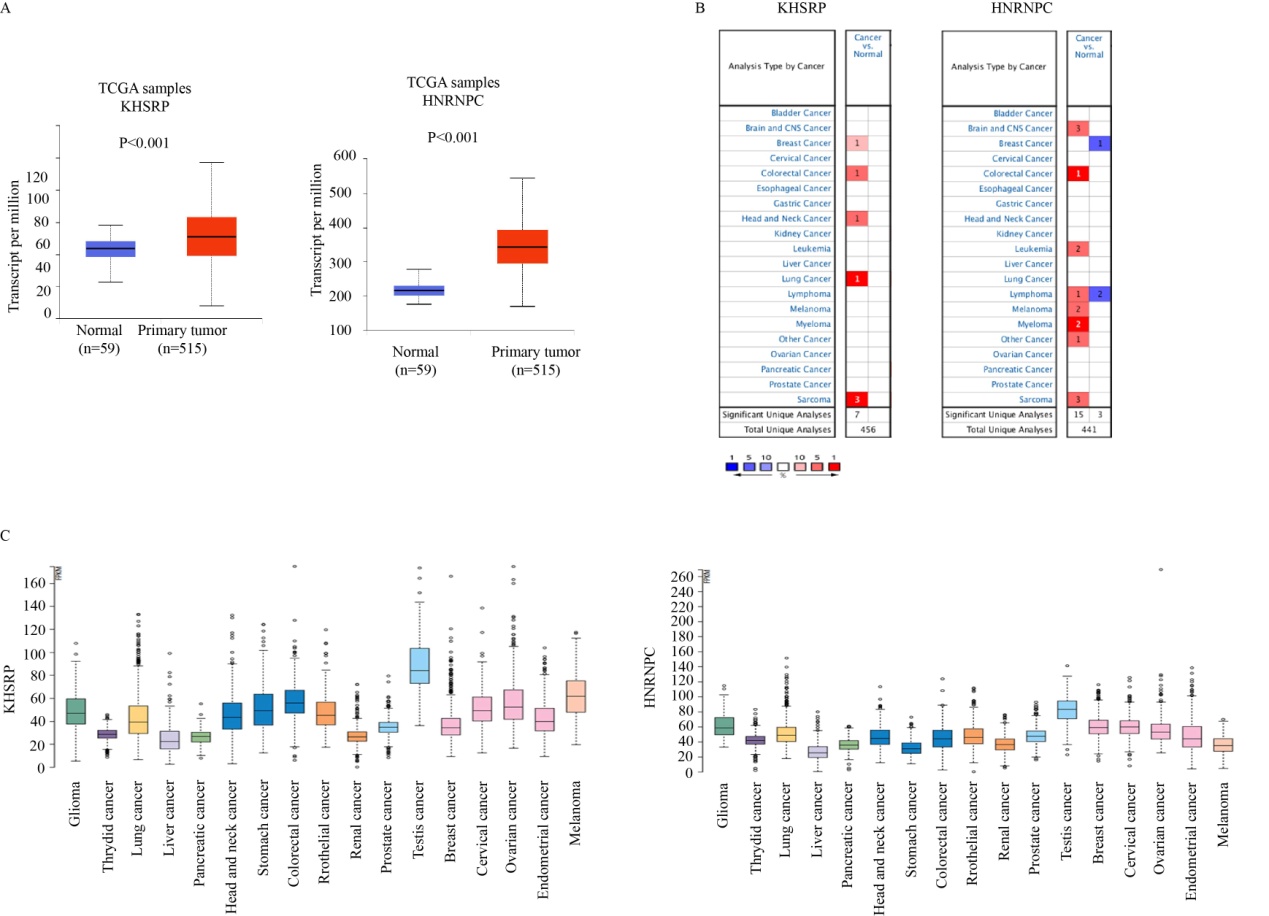


Supplementary figure 6


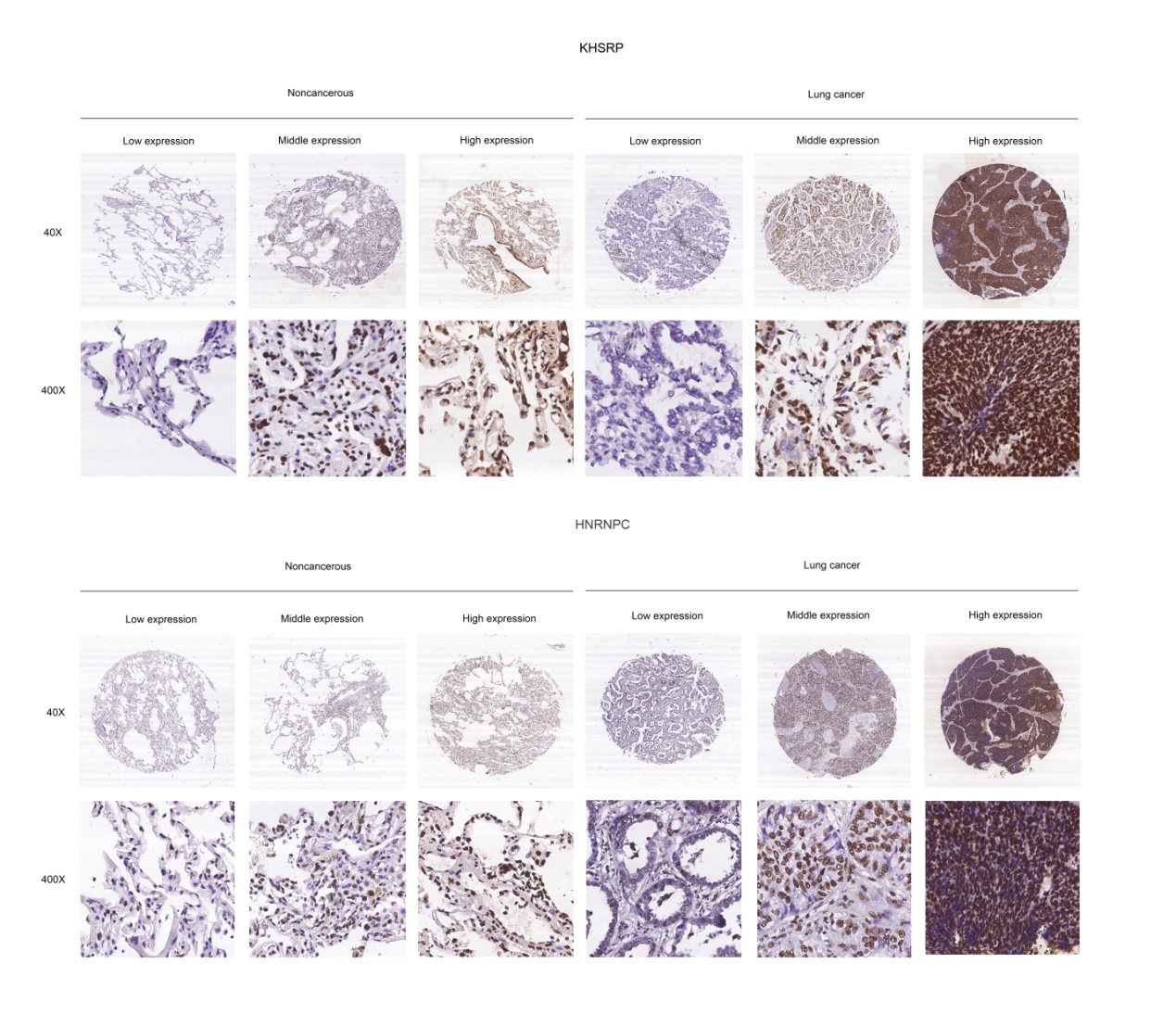


Supplementary figure 7


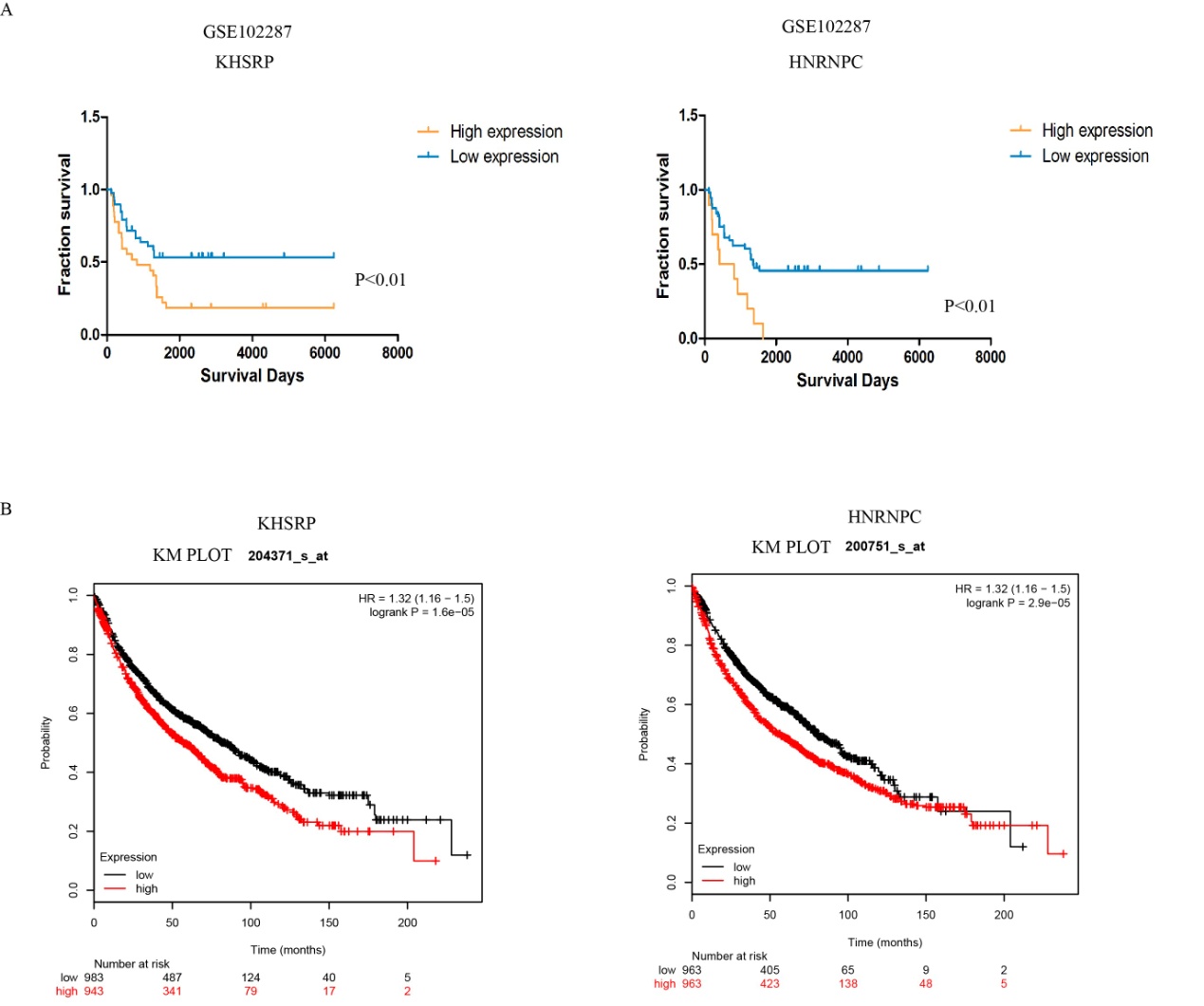


Supplementary Table 1. Primer sequences for real-time PCR used in the study

| Primer name | Primer sequences | |
| --- | --- | --- |
| KHSRP | sense | 5’-GCAGCAGGCTCAATGAATCG-3’ |
|  | antisense | 5’-GTGAGACACAGAACAGGCGA-3’ |
| HNRNPC | sense | 5’-TATTGCTCGGGCTGTAGTGC-3’ |
|  | antisense | 5’-ATCCCCGCTGTCCACTCTTA-3’ |
| β-actin | sense | 5’-ACAGGGAAAAGATGACACAGATCA-3’ |
|  | antisense | 5’-CAGCCTGGATGGCAACGTA-3’ |

Supplementary Table 2. Primer sequences for siRNA used in the study

| Primer name | Primer sequences | |
| --- | --- | --- |
| Hs-KHSRP-sh | sense | 5’-GCCUACUACUCACACUACUdTdT-3’ |
|  | antisense | 5’-AGUAGUGUGAGUAGUAGGCdTdT-3’ |
| Hs-HNRNPC-si-1 | sense | 5’-GGGACUAUUAUGAUAGGAUdTdT-3’ |
|  | antisense | 5’-AUCCUAUCAUAAUAGUCCCdTdTdTdT-3’ |
| Hs-HNRNPC-si-2 | sense | 5’-CCUUCGUUCAGUAUGUUAAdTdT-3’ |
|  | antisense | 5’-UUAACAUACUGAACGAAGGdTdT-3’ |
| Hs-HNRNPC-si-3 | sense | 5’-GCGUGUAUCAGGAAACACUdTdT-3’ |
|  | antisense | 5’-AGUGUUUCCUGAUACACGCdTdT-3’ |

Supplementary Table 3. The 52 up-regulated differential expression proteins identified by iTraq and SWATH^TM^ two proteomics methods

| Accession # | iTRAQ | | | | |  | SWATH^TM^ | |
| --- | --- | --- | --- | --- | --- | --- | --- | --- |
|  | Unused | % Cov | Peptides | H1299: H358 | *P*-value |  | Fold Change | *P*-value |
| sp|A6NHR9|SMHD1 | 75.67 | 45.1 | 46 | 2.7542 | 0.0000 |  | 2.4552 | 0.0000 |
| sp|O14776|TCRG1 | 52.34 | 37.1 | 28 | 2.6062 | 0.0000 |  | 4.1182 | 0.0020 |
| sp|O60841|IF2P | 47.24 | 42.1 | 31 | 2.6792 | 0.0001 |  | 2.3405 | 0.0007 |
| sp|O75475|PSIP1 | 39.81 | 54 | 32 | 3.5645 | 0.0000 |  | 2.4266 | 0.0000 |
| sp|O95239|KIF4A | 53.57 | 49.2 | 38 | 2.421 | 0.0000 |  | 1.8798 | 0.0005 |
| sp|P04406|G3P | 53.63 | 87.2 | 70 | 2.884 | 0.0002 |  | 1.5030 | 0.0000 |
| sp|P06493|CDK1 | 16.82 | 59.9 | 14 | 2.6792 | 0.0176 |  | 1.7218 | 0.0079 |
| sp|P06748|NPM | 61 | 78.6 | 119 | 4.0551 | 0.0002 |  | 1.5696 | 0.0002 |
| sp|P08670|VIME | 61.73 | 72.8 | 64 | 32.2107 | 0.0000 |  | 24.6525 | 0.0000 |
| sp|P14866|HNRPL | 84.16 | 80.3 | 133 | 4.4055 | 0.0000 |  | 1.9700 | 0.0000 |
| sp|P24534|EF1B | 24.6 | 81.8 | 42 | 1.803 | 0.0063 |  | 1.8648 | 0.0004 |
| sp|P26599-3|PTBP1 | 85.24 | 87.4 | 112 | 2.9648 | 0.004 |  | 4.7978 | 0.0003 |
| sp|P26641|EF1G | 53.98 | 76.2 | 60 | 2.4434 | 0.0001 |  | 2.1422 | 0.0000 |
| sp|P35659|DEK | 29.02 | 60.3 | 26 | 2.6792 | 0.0002 |  | 1.9698 | 0.0002 |
| sp|P42166|LAP2A | 67.01 | 78.4 | 61 | 3.6308 | 0.0001 |  | 3.1108 | 0.0005 |
| sp|P43358|MAGA4 | 7.71 | 30 | 5 | 8.2414 | 0.0013 |  | 10.3331 | 0.0001 |
| sp|P46013|KI67 | 114.94 | 47.5 | 74 | 10.2802 | 0.0000 |  | 3.0178 | 0.0003 |
| sp|P46063|RECQ1 | 19.59 | 36.7 | 16 | 8.7902 | 0.0000 |  | 2.4414 | 0.0035 |
| sp|P50552|VASP | 18.6 | 41.6 | 11 | 2.8576 | 0.0042 |  | 2.1984 | 0.0000 |
| sp|P52292|IMA1 | 49.66 | 76.6 | 39 | 1.7378 | 0.0001 |  | 1.7544 | 0.0002 |
| sp|P62937|PPIA | 51.05 | 91.5 | 91 | 9.1201 | 0.0002 |  | 2.0040 | 0.0000 |
| sp|P78347|GTF2I | 90.13 | 64.9 | 76 | 2.1878 | 0.0000 |  | 2.0673 | 0.0005 |
| sp|Q12769|NU160 | 24.38 | 28 | 16 | 2.4889 | 0.001 |  | 1.5236 | 0.0120 |
| sp|Q13573|SNW1 | 27.17 | 57.3 | 23 | 2.9107 | 0.0005 |  | 2.6435 | 0.0004 |
| sp|Q14204|DYHC1 | 304.25 | 59.5 | 276 | 3.2509 | 0.0000 |  | 1.6394 | 0.0003 |
| sp|Q14676|MDC1 | 76.18 | 48.8 | 54 | 5.2481 | 0.0002 |  | 3.3161 | 0.0000 |
| sp|Q14683|SMC1A | 100.35 | 61.5 | 75 | 4.0551 | 0.0000 |  | 1.8154 | 0.0002 |
| sp|Q15050|RRS1 | 15.07 | 48.2 | 12 | 4.0551 | 0.0022 |  | 3.0324 | 0.0000 |
| sp|Q15691|MARE1 | 24.93 | 67.5 | 29 | 2.1878 | 0.0041 |  | 2.0395 | 0.0024 |
| sp|Q29RF7|PDS5A | 57.24 | 45.5 | 44 | 12.7057 | 0.0000 |  | 1.5116 | 0.0103 |
| sp|Q2TAY7|SMU1 | 18.33 | 33.7 | 15 | 2.0324 | 0.0406 |  | 2.2629 | 0.0004 |
| sp|Q6NZI2|PTRF | 16.01 | 50.3 | 14 | 7.1121 | 0.0015 |  | 7.0589 | 0.0026 |
| sp|Q8IY18|SMC5 | 24.76 | 33.4 | 15 | 2.8576 | 0.0004 |  | 2.5595 | 0.0103 |
| sp|Q8NFW8|NEUA | 17.72 | 46.5 | 10 | 5.4954 | 0.0015 |  | 1.8186 | 0.0063 |
| sp|Q92878|RAD50 | 60.69 | 51.4 | 43 | 3.8726 | 0.0000 |  | 1.7556 | 0.0053 |
| sp|Q92945|FUBP2 | 68.77 | 71.2 | 75 | 1.9953 | 0.0007 |  | 1.5206 | 0.0000 |
| sp|Q92979|NEP1 | 9.39 | 44.3 | 6 | 6.4269 | 0.0138 |  | 2.8541 | 0.0000 |
| sp|Q96T88|UHRF1 | 29.1 | 43.3 | 24 | 2.0137 | 0.0013 |  | 1.9929 | 0.0034 |
| sp|Q99986|VRK1 | 26.93 | 60.9 | 24 | 3.3419 | 0.0002 |  | 2.0828 | 0.0005 |
| sp|Q9BQG0|MBB1A | 44.3 | 41.4 | 39 | 2.4434 | 0.0000 |  | 2.9882 | 0.0000 |
| sp|Q9BVJ6|UT14A | 15.15 | 34.4 | 9 | 1.8707 | 0.0074 |  | 1.9678 | 0.0007 |
| sp|Q9H307|PININ | 18.26 | 40.3 | 12 | 1.8707 | 0.0008 |  | 1.6633 | 0.0007 |
| sp|Q9NR30|DDX21 | 61.71 | 56.5 | 49 | 6.792 | 0.0000 |  | 1.9637 | 0.0000 |
| sp|Q9NU22|MDN1 | 90.71 | 30.7 | 63 | 3.9084 | 0.0000 |  | 3.3726 | 0.0091 |
| sp|Q9P2N5|RBM27 | 24.11 | 36.3 | 19 | 2.6546 | 0.0008 |  | 1.7214 | 0.0027 |
| sp|Q9UBD5|ORC3 | 14.33 | 26.4 | 11 | 10 | 0.0003 |  | 3.8301 | 0.0032 |
| sp|Q9Y5K6|CD2AP | 16.45 | 31.6 | 9 | 3.8726 | 0.0071 |  | 3.2275 | 0.0039 |
| tr|A4D2Q0|A4D2Q0 N | 27.09 | 47.1 | 15 | 4.1687 | 0.0004 |  | 2.0543 | 0.0229 |
| tr|B0V043|B0V043 N | 55.18 | 42.3 | 36 | 2.8314 | 0.0000 |  | 1.5069 | 0.0008 |
| tr|D6RBZ0|D6RBZ0 N | 34.43 | 69.7 | 46 | 4.1305 | 0.0013 |  | 1.7196 | 0.0000 |
| tr|E9PAU2|E9PAU2 N | 26.04 | 41.7 | 22 | 1.7219 | 0.0210 |  | 2.2808 | 0.0002 |
| tr|I3L3A8|I3L3A8A | 28.33 | 40.7 | 20 | 2.8314 | 0.0065 |  | 1.5953 | 0.0035 |

Supplementary Table 4. The 64 down-regulated differential expression proteins identified by iTraq and SWATH^TM^ two proteomics methods

| Accession # | iTRAQ | | | | |  | SWATH^TM^ | |
| --- | --- | --- | --- | --- | --- | --- | --- | --- |
|  | Unused | % Cov | Peptides | H1299: H358 | *P*-value |  | Fold Change | *P*-value |
| sp|O00231|PSD11 | 22.52 | 46.2 | 15 | 0.2377 | 0.0033 |  | 0.1795 | 0.0033 |
| sp|O14818|PSA7 | 16.6 | 56.5 | 11 | 0.2805 | 0.0073 |  | 0.1316 | 0.0003 |
| sp|O15355|PPM1G | 8.7 | 30 | 7 | 0.3837 | 0.0365 |  | 0.3198 | 0.0016 |
| sp|O43707|ACTN4 | 94.33 | 72.8 | 94 | 0.4529 | 0.0000 |  | 0.5461 | 0.0002 |
| sp|O43719|HTSF1 | 24.2 | 64.5 | 25 | 0.207 | 0.0002 |  | 0.1841 | 0.0014 |
| sp|O60264|SMCA5 | 63.77 | 52.2 | 40 | 0.6486 | 0.0474 |  | 0.5848 | 0.0004 |
| sp|O75083|WDR1 | 19.16 | 43.2 | 13 | 0.4529 | 0.0204 |  | 0.5641 | 0.0043 |
| sp|P04083|ANXA1 | 31.91 | 72.8 | 30 | 0.0946 | 0.0000 |  | 0.1287 | 0.0000 |
| sp|P05387|RLA2 | 19.78 | 85.2 | 18 | 0.1977 | 0.0040 |  | 0.5494 | 0.0000 |
| sp|P05455|LA | 23.41 | 55.2 | 16 | 0.2704 | 0.0000 |  | 0.3294 | 0.0036 |
| sp|P06733|ENOA | 61.5 | 83.6 | 92 | 0.092 | 0.0000 |  | 0.4500 | 0.0000 |
| sp|P07237|PDIA1 | 42.99 | 70.7 | 32 | 0.118 | 0.0000 |  | 0.2825 | 0.0315 |
| sp|P07355-2|ANXA2 | 73.03 | 86 | 109 | 0.0973 | 0.0000 |  | 0.3183 | 0.0000 |
| sp|P08727|K1C19 | 44.83 | 81 | 39 | 0.047 | 0.0023 |  | 0.0149 | 0.0000 |
| sp|P08729|K2C7 | 38.28 | 71.6 | 50 | 0.0288 | 0.0000 |  | 0.0029 | 0.0000 |
| sp|P08758|ANXA5 | 25.83 | 68.8 | 19 | 0.0855 | 0.0000 |  | 0.3219 | 0.0000 |
| sp|P11021|GRP78 | 52.84 | 68.8 | 63 | 0.1047 | 0.0000 |  | 0.1709 | 0.0000 |
| sp|P11216|PYGB | 44.03 | 52.9 | 34 | 0.1754 | 0.0000 |  | 0.1143 | 0.0001 |
| sp|P12277|KCRB | 16.67 | 46.5 | 10 | 0.1486 | 0.0000 |  | 0.1784 | 0.0003 |
| sp|P14618|KPYM | 61.46 | 73.3 | 60 | 0.2188 | 0.0000 |  | 0.4281 | 0.0007 |
| sp|P14625|ENPL | 31.82 | 46 | 30 | 0.1738 | 0.0001 |  | 0.4026 | 0.0075 |
| sp|P14923|PLAK | 37.57 | 54 | 24 | 0.1096 | 0.0001 |  | 0.0240 | 0.0000 |
| sp|P17980|PRS6A | 33.24 | 65.2 | 24 | 0.3311 | 0.0000 |  | 0.5685 | 0.0410 |
| sp|P19338|NUCL | 63.82 | 52.5 | 56 | 0.3436 | 0.0000 |  | 0.4188 | 0.0000 |
| sp|P21589|5NTD | 24.61 | 47.7 | 16 | 0.5808 | 0.0452 |  | 0.5503 | 0.0017 |
| sp|P23284|PPIB | 25.34 | 60.2 | 29 | 0.2188 | 0.0000 |  | 0.4463 | 0.0000 |
| sp|P28066|PSA5 | 14.22 | 50.2 | 11 | 0.1871 | 0.0023 |  | 0.0739 | 0.0002 |
| sp|P30101|PDIA3 | 37.03 | 72.5 | 31 | 0.1585 | 0.0000 |  | 0.2869 | 0.0000 |
| sp|P33991|MCM4 | 54.38 | 57.9 | 37 | 0.2249 | 0.0000 |  | 0.2678 | 0.0000 |
| sp|P33993|MCM7 | 39.31 | 54.9 | 30 | 0.4613 | 0.0001 |  | 0.4537 | 0.0439 |
| sp|P35221|CTNA1 | 47.66 | 51.9 | 41 | 0.1629 | 0.0000 |  | 0.2869 | 0.0001 |
| sp|P35579|MYH9 | 211.04 | 71.2 | 244 | 0.3162 | 0.0000 |  | 0.3096 | 0.0000 |
| sp|P35998|PRS7 | 20.11 | 50.1 | 14 | 0.1837 | 0.0000 |  | 0.3738 | 0.0186 |
| sp|P37802|TAGL2 | 24.15 | 76.4 | 31 | 0.0425 | 0.0000 |  | 0.1056 | 0.0000 |
| sp|P46777|RL5 | 15.65 | 52.5 | 13 | 0.2992 | 0.0018 |  | 0.5397 | 0.0003 |
| sp|P49327|FAS | 78.82 | 42.1 | 51 | 0.1514 | 0.0000 |  | 0.2038 | 0.0003 |
| sp|P49736|MCM2 | 50.74 | 54.8 | 39 | 0.2729 | 0.0000 |  | 0.2104 | 0.0000 |
| sp|P51858|HDGF | 35.65 | 79.6 | 36 | 0.1514 | 0.0002 |  | 0.4758 | 0.0002 |
| sp|P55072|TERA | 57.8 | 65.8 | 50 | 0.3133 | 0.0000 |  | 0.3942 | 0.0000 |
| sp|P55145|MANF | 14.39 | 63.2 | 15 | 0.2704 | 0.0008 |  | 0.4411 | 0.0000 |
| sp|P60903|S10AA | 7.87 | 90.7 | 10 | 0.1419 | 0.0190 |  | 0.4032 | 0.0000 |
| sp|P61201|CSN2 | 12.17 | 39.1 | 8 | 0.4875 | 0.0353 |  | 0.1177 | 0.0032 |
| sp|P62333|PRS10 | 18.11 | 54.5 | 10 | 0.2208 | 0.0073 |  | 0.2584 | 0.0004 |
| sp|Q08AF3|SLFN5 | 12.82 | 30.1 | 10 | 0.1148 | 0.0021 |  | 0.0360 | 0.0000 |
| sp|Q13200|PSMD2 | 30.38 | 38.7 | 17 | 0.227 | 0.0000 |  | 0.2898 | 0.0000 |
| sp|Q14126|DSG2 | 28.18 | 30.2 | 21 | 0.2421 | 0.0006 |  | 0.1463 | 0.0002 |
| sp|Q14566|MCM6 | 35.09 | 52.4 | 26 | 0.2355 | 0.0000 |  | 0.3272 | 0.0085 |
| sp|Q14764|MVP | 49.64 | 58.3 | 33 | 0.0649 | 0.0000 |  | 0.0884 | 0.0000 |
| sp|Q15149-4|PLEC | 259.92 | 61.8 | 185 | 0.1 | 0.0000 |  | 0.1399 | 0.0000 |
| sp|Q16666|IF16 | 51.1 | 52.6 | 39 | 0.1393 | 0.0006 |  | 0.0565 | 0.0000 |
| sp|Q86XP3|DDX42 | 46.34 | 54.6 | 28 | 0.2606 | 0.0024 |  | 0.4366 | 0.0004 |
| sp|Q8N163|K1967 | 50.41 | 61 | 40 | 0.3436 | 0.0000 |  | 0.3650 | 0.0000 |
| sp|Q99460|PSMD1 | 37.01 | 46.1 | 26 | 0.2512 | 0.0000 |  | 0.1345 | 0.0059 |
| sp|Q9NR12|PDLI7 | 12.07 | 37.6 | 6 | 0.4325 | 0.0188 |  | 0.5349 | 0.0003 |
| sp|Q9P258|RCC2 | 26.09 | 49.8 | 19 | 0.5916 | 0.0107 |  | 0.4497 | 0.0212 |
| sp|Q9Y446|PKP3 | 20.12 | 40.5 | 14 | 0.0461 | 0.0036 |  | 0.0497 | 0.0001 |
| tr|B4DGP8|B4DGP8 | 18.29 | 33.3 | 14 | 0.4446 | 0.0128 |  | 0.1498 | 0.0000 |
| tr|B4DWW4|B4DWW4 | 49.54 | 56.4 | 38 | 0.3251 | 0.0001 |  | 0.3612 | 0.0001 |
| tr|E7EU96|E7EU96 | 20.88 | 50.9 | 18 | 0.5754 | 0.0190 |  | 0.3768 | 0.0000 |
| tr|E9PK25|E9PK25 | 33.69 | 83.3 | 28 | 0.1837 | 0.0020 |  | 0.5408 | 0.0000 |
| tr|G3XAD8|G3XAD8 | 18.22 | 52.7 | 19 | 0.3664 | 0.0024 |  | 0.6658 | 0.0047 |
| tr|H0Y4R1|H0Y4R1 | 19.06 | 37.5 | 9 | 0.5754 | 0.0129 |  | 0.3616 | 0.0013 |
| tr|J3KPF3|J3KPF3 | 24.95 | 38.4 | 15 | 0.1343 | 0.0000 |  | 0.2800 | 0.0003 |
| tr|Q4JM47|Q4JM47 | 8.96 | 59.5 | 10 | 0.0449 | 0.0013 |  | 0.0177 | 0.0000 |
